# Advancing Glucose Conjugated Gibberellins Discovery: A Structure–Oriented Screening and Identification Method for Unraveling Gibberellin Metabolites in Plants

**DOI:** 10.3390/metabo14020096

**Published:** 2024-01-29

**Authors:** Chen Zeng, Wen-Jing Cai, Liu-Cheng Jiang, Tiantian Ye, Yu-Qi Feng

**Affiliations:** 1 School of Bioengineering and Health, Wuhan Textile University, Wuhan 430200, China; zengchen3351@whu.edu.cn; 2 Department of Chemistry, Wuhan University, Wuhan 430072, China; 2010301040094@whu.edu.cn (W.-J.C.); jianglc@whu.edu.cn (L.-C.J.); 3 School of Public Health, Wuhan University, Wuhan 430071, China; 4 Frontier Science Center for Immunology and Metabolism, Wuhan University, Wuhan 430071, China

**Keywords:** glucose conjugated gibberellins, isotope labeling, liquid chromatography, mass spectrometry fragmentation, quantitative structure–retention relationship

## Abstract

Gibberellins (GAs) play a pivotal role in modulating plant growth and development. Glucose–conjugated gibberellins (Glc–GAs), a prevalent conjugated form of GAs, regulate intracellular GA levels by the coupling and decoupling of glucose groups. However, the diversity of Glc–GAs identified within individual species remains limited, hinting at a multitude of yet undiscovered gibberellin metabolites. This lacuna poses considerable impediments to research efforts dedicated to comprehensively delineating the GA metabolic pathway. In this study, we developed a structure–oriented screening and identification method for Glc–GAs in plant species by employing LC–MS/MS coupled with chemical derivatization. Through the application of chemical derivatization technique, carboxyl groups on Glc–GAs were labeled which effectively enhanced the sensitivity and selectivity of mass spectrometry detection for these compounds. Concurrently, the integration of mass spectrometry fragmentation and chromatographic retention behavior facilitated the efficient screening and identification of potential Glc–GAs. With this strategy, we screened and identified 12 potential Glc–GAs from six plant species. These findings expand the Glc–GA diversity in plants and contribute to understanding GA metabolic pathways.

## 1. Introduction

Gibberellins (GAs) are carboxyl–containing plant hormones characterized by a tetracyclic diterpene skeleton. They are closely involved in various plant growth and development processes, including seed germination, hypocotyl elongation, and control of flowering time [1,2]. During the physiological regulation of GAs, free GAs can reversibly bind to small molecular groups through processes like glycosylation, acylation, or esterification, forming conjugated GAs [3,4]. Conjugated GAs also play important roles, mainly by altering the levels of GAs in the plant and regulating the endogenous GA reservoir through the binding and unbinding of conjugated groups [5,6]. During seed germination or plant growth periods, free GAs can be released from conjugated GAs through enzymatic hydrolysis to exert their effects, whereas, in mature plants, free GAs are converted into conjugated GAs and stored, which may be a crucial step in further degradation and metabolism processes [7,8,9]. Moreover, considering the polar properties of some conjugated GAs, they are also believed to be involved in intracellular compartmentalized transport and long–distance transport throughout the plant [10,11]. Therefore, conjugated GAs commonly serve as storage compounds, facilitators of transport, and intermediates in breakdown metabolism, playing an important role in the regulation of GA homeostasis.

Glucose derivatives are the most common conjugated GAs in plants [12]. Glycosylation is a component of both primary and secondary metabolism in plant tissues, and plants can maintain cellular homeostasis by regulating the level, biological activity, and subcellular distribution of glycosylated compounds [3,4,13]. It has been suggested that the polar glucose moiety in some conjugated GAs may cause a twisted orientation of the gibberellin molecule in the membrane, thereby preventing its binding to specific receptors [13,14]. Since the first gibberellin glucoside, GA_8_–2–*O*–glucoside, was isolated from mature beans, researchers have identified a series of GA glucose derivatives. Glucose–conjugated gibberellins (Glc–GAs) are mainly divided into two categories: one is the connection of glucose to the hydroxyl group on the GA skeleton, called GA glucosides, in which the glucose component can be connected to the carbon 2–*O*, 3–*O*, 11–*O*, 13–*O*, or 17–*O* position of the parent GA, generating a series of isomers. The other is the connection of glucose to the carboxyl group at the C–6 position of the GA skeleton, called GA glucosyl esters [7]. GA glucosyl esters are widely distributed in legumes, and their contents are relatively high in mature seeds [15]. In plants, free GAs are synthesized into gibberellin glucosides and glucosyl esters by glycosyltransferases to reduce the level of free GAs. Glycosyltransferase from mature bean fruit tissue can specifically glycosylate GA_3_, forming GA_3_–3–*O*–glucoside using UDP–glucose as the glucose donor [15]. Piccoli et al. found rapid conversion among GA glucosides, GA glucosyl esters, and free GAs in maize seedlings [16]. Thus, the presence of conjugated gibberellins can effectively maintain GA homeostasis and prevent plants from synthesizing free GAs de novo, which plays a crucial role in the dynamic balance of plant hormones.

As derivatives of free GAs, Glc–GAs typically exist in trace amounts in plant tissues [17]. Analyzing these compounds has been challenging due to their lack of luminescent or chromophoric groups and the complex plant matrix in which they reside [3]. Research on Glc–GAs has mainly focused on the period from the 1960s to the 1990s, during which researchers usually employed activity–directed screening strategies combined with GC–MS analysis to discover and identify Glc–GAs in plant samples [18,19]. Typically, a large amount of plant material is extracted with organic solvents, followed by liquid–liquid extraction for preliminary purification. The resulting fractions obtained from HPLC fractionation were subjected to activity assays, and the active fractions were subsequently analyzed by GC–MS and compared with synthetic standards to obtain qualitative results [18,20,21,22]. This approach is time–consuming, requires large quantities of material, and may overlook the majority of inactive or low–activity Glc–GAs. As a result, the variety of Glc–GAs found in plants is very limited. Compared to the over one hundred species of free–form GAs currently discovered in various organisms, the identified types of conjugated GAs are extremely limited. According to our literature survey, 20 species of conjugated GAs have been reported (Table S1). However, there is still a lack of efficient screening and identification methods for low–content and inactive or low–activity Glc–GAs [23]. Additionally, there is still little knowledge about the mechanism of action of glycosyltransferases in biology, and the relevant genes expressing glycosyltransferases have not been identified. Therefore, achieving accurate and quantitative analysis of trace Glc–GAs will contribute to the discovery of new Glc–GAs in plants and the study of their regulatory mechanism.

In previous studies, we developed a chemically assisted LC–MS technique for GA screening, which had the advantages of strong structure orientation, high sensitivity, and the ability to detect more low–activity GAs [24]. Building upon this, we established a method that combines this technique with structure–oriented Glc–GA screening and identification. By using chemical derivatization to enhance the mass spectrometric detection sensitivity of Glc–GAs, we were able to screen for potential Glc–GAs at low concentrations. Furthermore, the labeled Glc–GA molecules exhibited characteristic fragmentation patterns that could be used for structural inferences, assisting in the identification of potential Glc–GA structures. Ultimately, we screened and identified 12 potential Glc–GAs in six plant samples. This work is of great significance in improving our understanding of GA metabolic pathways and investigating the mechanisms of GA function.

## 2. Materials and Methods

### 2.1. Chemicals and Materials

Gibberellin standard GA_3_ was purchased from Olchemim Co., Ltd. (Olomouc, Czech Republic). Ammonia solution (25%), hydrochloric acid (HCl), and sodium chloride (NaCl) were obtained from Sinopharm Chemical Reagent Co., Ltd. (Shanghai, China). α–D–bromotetraacetylglucopyranose (98%), silver oxide (Ag_2_O, 99.7%), and sodium methoxide (NaOMe, 97%) were purchased from Aladdin Reagent Co., Ltd. (Shanghai, China). 4A molecular sieves were obtained from Macklin Reagent Co., Ltd. (Shanghai, China). Chromatography–grade methanol (MeOH) was purchased from Merck Reagent Co., Ltd. (Darmstadt, Germany). Dichloromethane (99.9%, anhydrous solvent with molecular sieves) was acquired from J&K Scientific Co., Ltd. (Beijing, China). Analytical–grade formic acid (FA, 88%), 2–chloro–1–methylpyridinium iodide (CMPI), triethylamine (TEA), and *N*,*N*–dimethylethylenediamine (DMED) were purchased from Sinopharm Chemical Reagent Co., Ltd. (Shanghai, China). The isotopically labeled reagent *d_4_*–DMED was synthesized following the reported methods in our laboratory [25]. Chromatography–grade acetonitrile (ACN) was obtained from Merck Reagent Co., Ltd. (Darmstadt, Germany). SAX–SPE (strong anion exchange–solid–phase extraction column, 3 mL, 200 mg) was purchased from Weltech Co., Ltd. (Wuhan, China). Milli–Q ultrapure water Co., Ltd. (Millipore, Bedford, USA) was used throughout the experiments. Stock solutions of TEA (10 μmol/mL), CMPI (20 μmol/mL), DMED (20 μmol/mL), *d_4_*–DMED (20 μmol/mL), and organic acids (1 mg/mL) were prepared in chromatography–grade ACN and stored at −20 °C in a refrigerator.

### 2.2. Collection of Plant Samples 

Mature seeds of *Canavalia gladiata*, *Phaseolus vulgaris*, *Pisum sativum*, and *Vicia faba* were purchased from local supermarkets in Wuhan, China. *Vigna angularis* was provided by the Vegetable Breeding Center of the Hubei Academy of Agricultural Sciences (Wuhan, China). Rice (*Oryza sativa* ssp. *japonica* cv. Nipponbare) samples were collected during the grain–filling stage, frozen in liquid nitrogen, and stored at −80 °C.

### 2.3. Extraction and Derivatization of Glc–GAs in Plant Samples 

Extraction of Glc–GAs from plant samples: 2 g of plant sample powder was weighed and mixed with 20 mL of MeOH (1 mL/100 mg) as the extraction solvent. The mixture was vortexed for 5 min and left to extract at 4 °C overnight. After centrifugation (5000 rpm, 5 min, 4 °C), the supernatant was collected and purified using SAX–SPE [26]. The purification process involved three steps: activation of the SPE column with 3 mL of MeOH, sample loading, and washing with 3 mL each of MeOH/H_2_O (1/9, *v*/*v*) and H_2_O/MeOH (1/9, *v/v*) solutions, followed by elution with 3 mL of the elution solution (MeOH containing 1% FA). The eluted fractions were collected and dried under nitrogen flow with heating at 40 °C.

The purified samples were reconstituted with 200 µL of ACN, divided into two portions, and each portion was separately added with 20 µL of TEA (10 μmol/mL) and CMPI (20 μmol/mL), followed by vortexing. To one portion, 20 µL of DMED (20 μmol/mL) was added, and to the other portion, 20 µL of *d_4_*–DMED (20 μmol/mL) was added. The derivatization reactions were conducted at 40 °C with shaking at 1500 rpm for 60 min. After completion of the derivatization, the samples were dried under nitrogen flow with heating at 40 °C. The light– and heavy–labeled samples were reconstituted with 100 µL of ACN/H_2_O (1/9, *v*/*v*) each and combined. The injection volume for subsequent LC–MS analysis was 10 µL.

### 2.4. Synthesis of Glc–GA_3_

First, 2 mmol of GA_3_ (693 mg) standard was weighed, and the GA_3_ was reacted with 3 mmol of derivatization reagent (TEA, CMPI, DMED) in a molar ratio of reactant to product of 1:1.5 under oscillation at 40 °C for 1 h. The reaction products were purified using a manually packed reverse–phase C18 column to remove excess derivatization reagents. The products were then placed in a 50 mL round–bottom flask and mixed with 1 g of α–D–bromotetraacetylglucopyranose, 1 g of Ag_2_O, 20 pellets of 4A molecular sieves (previously dried), and 20 mL of anhydrous dichloromethane. The mixture was magnetically stirred at 25 °C for 24 h. The reaction products were extracted with saturated NaCl solution, and the organic phase was dried and dissolved in 10% MeOH prior to mass spectrometry analysis. The software Chemdraw Professional 17.1 was used to predict the hydrophobicity of Glc–GA_3_ isomers.

### 2.5. Instrumentation and Analytical Conditions 

The instrument used for sample analysis was a UHPLC–MS system. The mass spectrometer employed was an Orbitrap Fusion Tribrid mass spectrometer (Thermo Fisher Scientific, Waltham, MA, USA). The liquid phase system consisted of an UltiMate 3000 UHPLC System (Thermo Fisher Scientific, USA). The chromatographic column used was a Waters ACQUITY UPLC BEH C18 Column (100 mm × 2.1 mm i.d., 1.7 μm, Waters, Milford, MA, USA), maintained at a column temperature of 40 °C. A 0.1% FA aqueous solution was used as mobile phase A and ACN as mobile phase B. The gradient elution for injection chromatography was as follows: 0–2 min, 5% B; 2–10 min, 5–15% B; 10–25 min, 15–50% B; 25–27 min, 50–90% B; 27–32 min, 90% B; 32–35 min, 90–5% B; 35–40 min, 5% B. The flow rate was set at 0.4 mL/min.

Data–dependent acquisition mode (DDA) was used to obtain mass spectrometry information. The DDA–HCD MS/MS data acquisition method employed the following parameters: FTMS (Full–MS): performed in positive ion mode, with a scan range of *m*/*z* 300–700; a resolution of 120,000; AGC target set to 2 × 105; precursor priority set to highest charge state and then most intense; FTMS2 (HCD): collision energy (%) at 50–60; a resolution of 30,000; ion source parameters: sheath gas flow at 35 psi, auxiliary gas at 15 psi, spray voltage at 3.5 kV, ion transfer capillary temperature at 320 °C, and heated block temperature at 350 °C.

The synthetic Glc–GA_3_ was detected in negative mode. The substances derived from DMED were detected in negative mode.

## 3. Results and Discussion

### 3.1. Strategies for Screening and Identifying Glc–GAs

**Figure 1:**
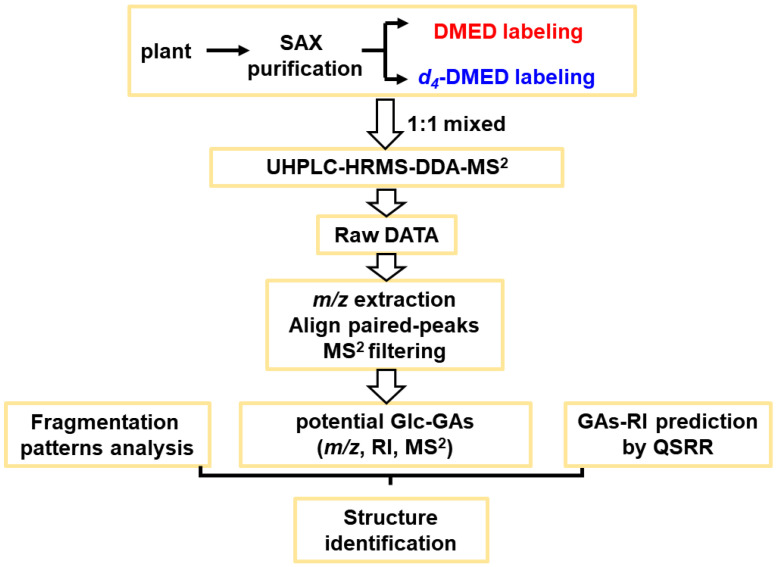
Overview of the workflow for the screening and identification of potential Glc–GAs from plant species.

**Figure 2:**
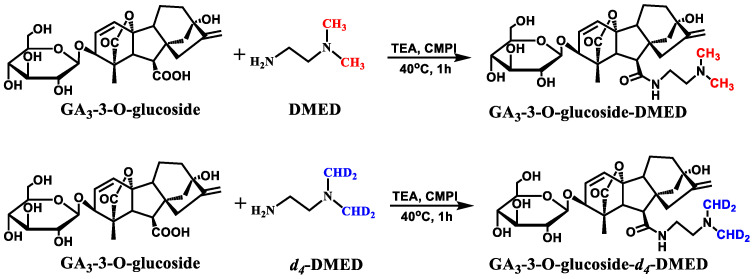
Derivatization of GA_3_–3–*O*–glucoside with DMED/*d*_4_–DMED.

Based on the structure–guided GA screening strategy previously reported [24], we established a screening method for Glc–GAs. This strategy utilized the mass spectrometry fragmentation behavior conferred by chemical derivatization to characterize Glc–GAs and screen for potential Glc–GA compounds. It combined mass spectrometry fragmentation and chromatographic retention behaviors for qualitative analysis and aided in structural inference. The specific workflow is illustrated in Figure 1. The plant samples were first pretreated for purification and enrichment. They were then reacted with a pair of derivatizing reagents (DMED/*d_4_*–DMED) according to the derivatization reaction equation shown in Figure 2. The labeled product was mixed in a 1:1 ratio and analyzed by UHPLC–MS to obtain the initial data. Table S2 lists the *m*/*z* values of labeled Glc–GA products for subsequent peak extraction and peak–pair matching. Additionally, the labeled Glc–GA products exhibited characteristic mass spectrometry fragmentation behavior. We employed five rules to screen the mass spectrometry spectra and identify potential Glc–GA candidates. Furthermore, we utilized mass fragmentation pathway analysis and quantitative structure–retention relationship models for structure elucidation, resulting in the final resolution of the structures.

### 3.2. Analysis of Mass Fragmentation Behavior of Glc–GAs–Labeled Products

Known GAs’ mass fragmentation behavior provides important clues for structure–oriented screening strategies. Our previous work has studied the fragmentation pathways of DMED–labeled products of existing GA standards, summarizing four distinctive fragmentation behaviors: (1) DMED–related fragmentation behavior; (2) GAs–A ring–related fragmentation behavior; (3) GAs–C–D ring–related fragmentation behavior; and (4) carbon skeleton–related fragmentation behavior [24]. These four characteristic fragmentation behaviors serve as important references for the analysis of the mass fragmentation behavior of Glc–GA labeled products.

Currently, Glc–GAs are not commercially available. In order to obtain Glc–GAs reference standards to explore the fragmentation patterns and retention time patterns using mass spectrometry, we attempted to synthesize Glc–GA_3_ following the methods described in references [10,27]. The structures of the substrate and potential products are shown in Figure S1. There are three possible Glc–GA_3_ main products: GA_3_–3–*O*–Glucoside, GA_3_–13–*O*–Glucoside, and GA_3_ glucosyl ester. Figure S2 shows the total ion chromatogram and extracted ion chromatograms (XIC) with *m*/*z* 507.1872 of the synthesized reaction products. From the XIC, it can be observed that there are three target product peaks with retention times of 8.78 min, 9.40 min, and 10.17 min, respectively. As shown in Figure S3, when observing the mass spectra at the retention times corresponding to the three product peaks, it is found that they exhibit a high degree of similarity in terms of fragment ion types. Among them, Figure S3A has noticeable differences in the relative intensities of the fragment ions compared to Figure S3B,C. 

Furthermore, by comparing the hydrophobicity constants predicted by the software for three Glc–GA_3_ compounds, namely GA_3_–3–*O*–Glucoside, GA_3_–13–*O*–Glucoside, and GA_3_ glucosyl ester, it was observed that their hydrophobicity decreases sequentially. As a result, their retention on the chromatographic column decreases, leading to shorter peak elution times. Based on the results of the secondary mass spectrometry (Figure S3), we speculated that the peak at 8.78 min corresponded to GA_3_ glucosyl ester, the peak at 9.40 min corresponds to GA_3_–13–*O*–Glucoside, and the peak at 10.17 min corresponds to GA_3_–3–*O*–Glucoside.

**Figure 3:**
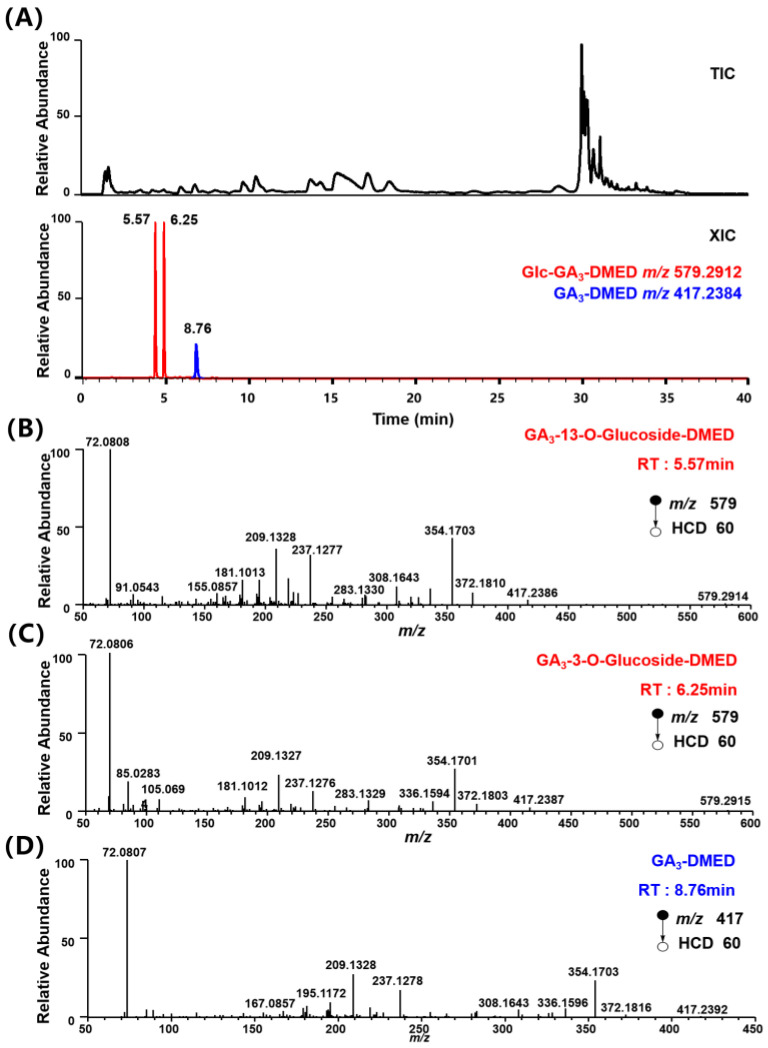
(**A**). Total ion chromatogram and extracted ion chromatograms of synthetic Glc–GA_3_ after DMED labeling (red: *m*/*z* 579.2912, blue: *m*/*z* 417.2384). HR MS/MS spectra of Glc–GA_3_–DMED (*m*/*z* 579) at (**B**) 5.57 min, (**C**) 6.25 min, GA_3_–DMED (*m*/*z* 417) at (**D**) 8.76 min under positive mode.

**Figure 4:**
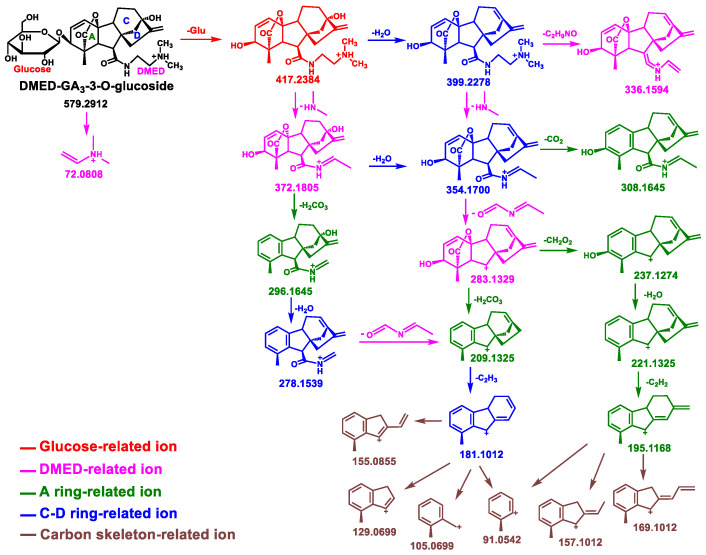
The proposed fragmentation pathways of DMED–GA_3_–3–*O*–Glucoside.

DMED can react with the carboxyl group of Glc–GA_3_. Taking GA_3_–3–*O*–glucoside as an example, Figure 2 shows the corresponding reaction equation. Figure 3A displays the total ion chromatogram (TIC) and extracted ion chromatograms (XIC) of the synthetic products after the chemical labeling reaction. In the XIC, the blue peak represents the unreacted GA_3_ labeled product at *m*/*z* 417.2384, while the red peak represents the Glc–GA_3_ labeled product at *m*/*z* 579.2912. Due to the carboxyl site of GA_3_ glucosyl ester being occupied by the glucose moiety and not reacting with DMED, there are only two red peaks with retention times of 5.57 min and 6.25 min. Considering their hydrophobicity, we speculated that the peak at 5.57 min corresponded to GA_3_–13–*O*–Glucoside–DMED and the peak at 6.25 min corresponded to GA_3_–3–*O*–Glucoside–DMED. By observing the mass spectra at the retention times corresponding to these peaks, it is found that, apart from the obvious Glucose–related fragmentation behaviors, the Glc–GA_3_–labeled products exhibit highly similar changes in fragment ion types and relative intensities compared to the substrate GA_3_ labeled products (Figure 3B–D). Figure 4 shows possible fragmentation pathways for DMED–GA_3_–3–*O*–glucoside. In addition to the four characteristic fragmentation behaviors mentioned above, Glc–GAs also exhibit Glucose–related fragmentation behaviors for losing glucose (162.0528), such as the protonated molecule *m*/*z* 579.2912 of GA_3_–3–*O*–glucoside fragmenting into the product ion *m*/*z* 417.2384. This fifth characteristic fragmentation behavior provides a further basis for inferring the structures of Glc–GAs. 

### 3.3. Establishment of Quantitative Structure–Retention Relationship (QSRR) Models

The Quantitative Structure–Retention Relationship (QSRR) model has gained significant attention in recent years. It associates the chromatographic retention properties of substances with their molecular structural features, enabling the prediction of compound retention behaviors and aiding in metabolite identification. In our previous research, we utilized a multivariate linear regression method to construct a QSRR model using a dataset of 13 GA standards. Through various cross–validation analyses, the model demonstrated good predictive ability and successfully predicted the retention index (RI) for 603 GA structures, with a root mean square value of 69.5832, which represents the threshold for matching experimental and predicted RIs [24].

In this research, we utilized the established QSRR model to assist in predicting the RI of potential Glc–GAs in our study. Glc–GAn exhibits a shorter retention time and lower RI compared to the corresponding GAn due to weaker glucose group retention on the C18 reversed–phase column. Figure 3A shows that the retention time of Glc–GA_3_ is earlier than that of GA_3_, and the RI of Glc–GA_3_ (575.25, 592.50) is smaller than that of GA_3_ (RI = 653.94). Therefore, considering the error threshold of the QSRR, the experimental RI value of potential Glc–GAn should be below the sum of the predicted RI value of GAn plus 69.5832 as shown in Equation (1), and we use this inference to facilitate the identification of potential Glc–GAn.

*RI_Glc__–__GAn_* < *Predicted RI_GAsn_* + 69.5832 (1)

### 3.4. Screening Potential Glc–GAs in Plant Samples

After overnight extraction of plant samples at a low temperature, they were purified using strong anion exchange solid–phase extraction columns. The samples were then divided into two portions and reacted separately with the derivatizing reagents DMED and *d_4_*–DMED. The labeled products were mixed in a 1:1 ratio and subjected to UHPLC–MS analysis to obtain initial data. Peak extraction was performed using the simulated *m*/*z* values in Table S2, followed by peak matching based on two criteria: (1) consistent retention time (RT_DMED–labeled_–RT*_d4_*_–DMED–labeled_: 0–0.013 min) and (2) consistent peak intensity (Intensity_DMED–labeled_/Intensity*_d4_*_–DMED–labeled_: 0.76–1.50). Peak pairs that satisfied both criteria were considered potential Glc–GA compounds.

**Figure 5:**
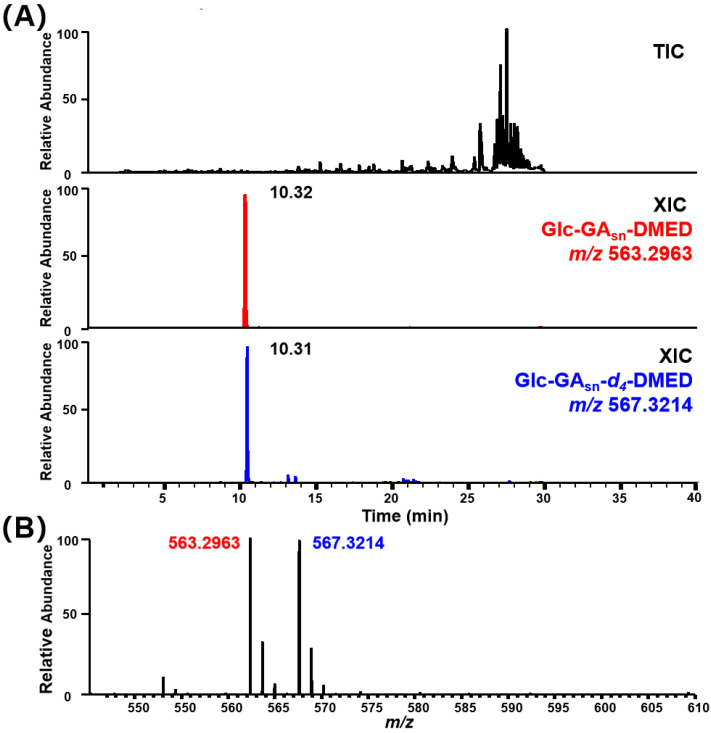
Chemical isotope–labeled *Canavalia gladiata* sample analyzed by LC–HRMS under full scan mode. (**A**) Total ion chromatogram of DMED/*d_4_*–DMED labeled *Canavalia gladiata* sample and extracted ion chromatograms of representative Glc–GA–DMED/*d_4_*–DMED channel at *m*/*z* 563.2963 and 567.3214. (**B**) Mass spectra at 10.32 min.

Taking sword bean samples as an example, Figure 5A shows the TIC and XIC plots of the labeled products in HRMS full scan mode, while Figure 5B provides information on the precursor ion peaks of the light and heavy labeled products. The peaks with *m*/*z* values of 563.2963 and 567.3214 exhibited consistent intensities and retention times, indicating that these compounds are potential Glc–GA compounds. As shown in Table S3, a total of 93 Glc–GA metabolites were detected in the six plant samples. Subsequently, we filtered the secondary mass spectra of 93 potential Glc–GAs using the aforementioned five categories of fragmentation patterns: (1) DMED–related fragmentation behaviors, such as the loss of (CH_3_)_2_NH (45.0578), C_4_H_10_N^+^ (72.0808), C_3_H_6_ON^+^ (72.0444), etc.; (2) GAs–A ring–related fragmentation behaviors, such as the loss of H_2_O (18.0106), HCOOH (46.1055), etc.; (3) GAs–C–D ring–related fragmentation behaviors, involving rearrangement via cleavage of C9–C11 and C8–C14 bonds, and loss of C_2_H_4_ (28.0313), etc.; (4) carbon backbone–related fragmentation behaviors, resulting in odd–numbered C_x_H_y_^+^ fragments in the *m*/*z* 80–230 range; (5) Glucose–related fragmentation behaviors, including loss of glucose (162.0528). Ultimately, we identified 12 Glc–GA candidates through this screening process. Their molecular weight information is provided in Table 1, and their MS/MS spectra are shown in Figures S4–S5, S9–S11, S13–S16, S18, S21, and S23.

### 3.5. Identification of Potential Glc–GAs in Plant Samples

After obtaining the 12 Glc–GA candidates, we performed structure identification through a four–step process. The first step involved determining the Glc–GA types corresponding to specific *m*/*z* values based on Table S2. In the second step, we analyzed the fragmentation pathways and filtered out Glc–GA candidates with inconsistent structures. The third step involved using a QSRR model to further eliminate Glc–GA candidates with mismatched retention indices (RI). In the fourth step, we compared the MS/MS spectra of the Glc–GA candidates with available standard GA compounds to obtain potential Glc–GA structures. Subsequently, we conducted an identification analysis on the screened potential Glc–GA compounds in the plant samples.

For compound **1** and compound **2**, the Glc–GA candidates to which the labeled product with *m*/*z* 563.2963 corresponds include Glc–GA_5_, Glc–GA_31_, Glc–GA_95_, Glc–GA_96_, Glc–GA_108_, Glc–GA_109_, Glc–GA_117_, Glc–GA_121_, Glc–GA_122_, Glc–GA_7_, Glc–GA_11_, Glc–GA_62_, Glc–GA_88_, Glc–GA_104_, Glc–GA_105_, Glc–GA_106_, and Glc–GA_107_ (Table S2). In the MS/MS spectrum of compound **1** (Figure S4), the labeled product with *m*/*z* 563.2963 sequentially loses glucose and (CH_3_)_2_NH to yield fragments with *m*/*z* 401.2418 and *m*/*z* 356.1840, respectively. Additionally, fragment ions with *m*/*z* 338.1737 and *m*/*z* 310.1791, derived from the loss of H_2_O and HCOOH from the fragment ion with *m*/*z* 356.1840 [M–Glucose–(CH_3_)_2_NH^+^], are observed. Similarly, in the MS/MS spectrum of compound **2** (Figure S5), the labeled product with *m*/*z* 563.2963 also exhibits a loss of glucose, (CH_3_)_2_NH, H_2_O, and HCOOH within the high mass range. Based on this information, it could be inferred that both compound **1** and compound **2** possessed a lactone and a hydroxyl group on the A–ring. Considering the structural features of the candidates, the possible structures were Glc–GA_7_, Glc–GA_62_, Glc–GA_88_, Glc–GA_104_, Glc–GA_105_, Glc–GA_106_, and Glc–GA_107_. The possible fragmentation pathways of Glc–GA_7_–DMED, Glc–GA_62_–DMED, Glc–GA_88_–DMED, Glc–GA_104_–DMED, Glc–GA_105_–DMED, Glc–GA_106_–DMED, and Glc–GA_107_–DMED are shown in Figure S6, which are consistent with the characteristic fragmentation behaviors of compound **1** and compound **2**, making it difficult to make a determination. To assist in the structural identification, a QSRR model was applied as a supplementary tool. From Table S4, the predicted retention indices (RIs) of GA_105_–DMED, GA_62_–DMED, GA_104_–DMED, GA_106_–DMED, GA_107_–DMED, GA_7_–DMED, and GA_88_–DMED are 535.52, 685.27, 716.94, 796.48, 826.57, 840.94, and 865.36, respectively. The differences between these predicted values and the experimental value of compound **1** (718.97) are −183.45, −33.70, −2.03, 77.51, 107.60, 121.97, and 146.39. Among them, the deviations of GA_62_–DMED, GA_104_–DMED, GA_106_–DMED, GA_107_–DMED, GA_7_–DMED, and GA_88_–DMED from the experimental value are greater than −69.58 but within the threshold of the prediction error of the model. The differences between these predicted values and the experimental value of compound **2** (760.66) are −225.14, −75.39, −43.72, 35.82, 65.91, 80.28, and 104.70. Among them, the deviations of GA_104_–DMED, GA_106_–DMED, GA_107_–DMED, GA_7_–DMED, and GA_88_–DMED from the experimental value are within the threshold of prediction error of the model. Furthermore, by comparing the secondary mass spectra of GA_7_ standards with compound **1** and compound **2** in plant samples, significant differences in the types and relative intensities of MS/MS fragment ions were observed (Figures S7 and S8). Therefore, compound **1** and compound **2** were not Glc–GA_7_. Based on the above analysis, the possible structures for compound **1** were Glc–GA_62_, Glc–GA_88_, Glc–GA_104_, Glc–GA_106_, or Glc–GA_107_, while the possible structures for compound **2** were Glc–GA_88_, Glc–GA_104_, Glc–GA_106_, or Glc–GA_107_.

For compounds **3**–**5**, Glc–GA candidates with *m*/*z* 565.3119 corresponding to the product ion include Glc–GA_20_, Glc–GA_69_, Glc–GA_70_, Glc–GA_84_, Glc–GA_4_, Glc–GA_40_, Glc–GA_51_, Glc–GA_61_, Glc–GA_119_, Glc–GA_45_, and Glc–GA_12_ (Table S2). The MS/MS spectra of compounds 3, 4, and 5 contain fragment ions *m*/*z* 340.1907, *m*/*z* 312.1963, and *m*/*z* 294.1852, generated by the consecutive loss of H_2_O and HCOOH from the [M–Glucose–(CH_3_)_2_NH^+^] fragment ion (Figures S9–S11), indicating the presence of a lactone and a hydroxyl group on ring A of compounds **3**–**5**. Based on the structural analysis of the candidates, possible structures were Glc–GA_4_, Glc–GA_40_, Glc–GA_51_, Glc–GA_61_, and Glc–GA_119_. Possible fragmentation pathways of Glc–GA_40_–DMED, Glc–GA_51_–DMED, Glc–GA_61_–DMED, Glc–GA_119_–DMED, and Glc–GA_4_–DMED are shown in Figure S12, all of which are positional isomers of glucose on ring A with similar fragmentation patterns. Considering the predicted retention indices (RI) (Table S4), it was speculated that the possible structures of compounds **3**–**5** were Glc–GA_4_, Glc–GA_40_, Glc–GA_51_, Glc–GA_61_, or Glc–GA_119_.

For compounds **6**–**9**, Glc–GA candidates with *m*/*z* 579.2912 corresponding to the product ion include Glc–GA_126_, Glc–GA_3_, Glc–GA_6_, Glc–GA_30_, Glc–GA_80_, Glc–GA_92_, Glc–GA_94_, Glc–GA_22_, and Glc–GA_68_ (Table S2). The MS/MS spectra of compounds **6-9** contain fragment ions *m*/*z* 354.1700, *m*/*z* 326.1751, and *m*/*z* 310.1802, generated by the consecutive loss of H_2_O and HCOOH from the [M–Glucose–(CH_3_)_2_NH^+^] fragment ion (Figures S13–S16), indicating the presence of a lactone, hydroxyl group, or epoxy group on ring A of compounds **6**–**9**. Based on the structural analysis of the candidates, possible structures were Glc–GA_3_, Glc–GA_6_, Glc–GA_30_, Glc–GA_80_, Glc–GA_92_, and Glc–GA_68_. Possible fragmentation pathways of Glc–GA_92_–DMED, Glc–GA_80_–DMED, Glc–GA_68_–DMED, Glc–GA_3_–DMED, Glc–GA_6_–DMED, and Glc–GA_30_–DMED are shown in Figure S17, which are consistent with the characteristic fragmentation behaviors of compounds **6**–**9**. Considering the predicted retention indices (RI) (Table S4) and the fragmentation pathway of Glc–GA_3_ (Figure 3), compound **6** would be Glc–GA_6_, Glc–GA_30_, Glc–GA_80_, or Glc–GA_68_; compound **7** would be Glc–GA_6_, Glc–GA_30_, Glc–GA_80_, or Glc–GA_68_; compound **8** would be Glc–GA_6_ or Glc–GA_30_; and compound **9** would be Glc–GA_6_ or Glc–GA_30_.

For compound **10**, the Glu–GA candidates corresponding to the marked product *m*/*z* 581.3068 include Glc–GA_77_, Glc–GA_130,_ Glc–GA_136_, Glc–GA_1_, Glc–GA_29_, Glc–GA_35_, Glc–GA_58_, Glc–GA_60_, Glc–GA_71_, Glc–GA_81_, Glc–GA_118_, Glc–GA_16_, Glc–GA_34_, Glc–GA_47_, Glc–GA_54_, Glc–GA_90_, Glc–GA_63_, Glc–GA_67_, and Glc–GA_131_ (Table S2). The MS/MS spectrum of compound **10** in the high–polymer region contains fragment ions generated by the consecutive loss of H_2_O, HCOOH, and CO_2_+2H_2_O from the [M–Glucose–(CH_3_)_2_NH^+^] fragment ion (374.1962) (Figure S18), indicating the presence of one lactone and two hydroxyl groups on the A–ring of compound **10**. Based on the structural characterization of the candidates, possible structures were Glc–GA_16_, Glc–GA_34_, Glc–GA_47_, Glc–GA_54_, and Glc–GA_90_. The potential fragmentation pathways of Glc–GA_90_–DMED, Glc–GA_16_–DMED, Glc–GA_54_–DMED, Glc–GA_47_–DMED, and Glc–GA_34_–DMED are shown in Figure S19, which are consistent with the characteristic fragmentation behaviors of compound **10**. After filtering through the QSRR model, the possible structures were Glc–GA_16_, Glc–GA_34_, Glc–GA_47_, and Glc–GA_54_ (Table S4). Comparison of the secondary mass spectra of standard GA_34_ with that of compound **10** (Figure S20) reveals similar types of ion fragments and relative intensity trends. Since the remaining candidates, Glc–GA_16_, Glc–GA_34_, Glc–GA_47_, and Glc–GA_54_, were isomers with glucose or hydroxyl groups on the A–ring and exhibited similar fragmentation behavior, further determination was not possible. Therefore, the possible structures for compound **10** were Glc–GA_16_, Glc–GA_34_, Glc–GA_47_, or Glc–GA_54_.

For compound **11**, the Glc–GA candidates corresponding to the marked product *m*/*z* 583.3225 include Glc–GA_2_ and Glc–GA_82_ (Table S2). In the MS/MS spectrum of compound **11**, the marked product *m*/*z* 583.3225 sequentially loses glucose and (CH_3_)_2_NH to obtain *m*/*z* 421.2697 and *m*/*z* 376.2118, respectively. Meanwhile, the fragment ion *m*/*z* 376.2118 exhibits the behavior of losing H_2_O and HCOOH (Figure S21), indicating the presence of one lactone and one hydroxyl group on the A–ring of compound **11**. Based on the structural analysis of the candidates, possible structures were Glc–GA_2_ and Glc–GA_82_. The potential fragmentation paths of Glc–GA_2_–DMED and Glc–GA_82_–DMED are shown in Figure S22, both of which are consistent with the characteristic fragmentation behaviors of compound **11**. Combining the results of the QSRR model filtering (Table S4), we speculated that the possible structures for compound **11** are Glc–GA_2_ or Glc–GA_82_.

For compound **12**, potential Glc–GA candidates corresponding to the marked product *m*/*z* 611.3174 include Glc–GA_125_, Glc–GA_129_, Glc–GA_23_, Glc–GA_52_, Glc–GA_13_, Glc–GA_17_, Glc–GA_46_, Glc–GA_66_, Glc–GA_99_ and Glc–GA_102_ (Table S2). The MS/MS spectrum of compound **12** includes a series of fragment ions in the higher mass region derived from the loss of 2H_2_O and HCOOH by the fragment ion [M–Glucose–(CH_3_)_2_NH^+^] (404.2068) (Figure S23), indicating the presence of one lactone and two hydroxyl groups on the A ring of compound **12**. Based on the structural analysis of the candidates, the possible structure was Glc–GA_52_. After filtering through the QSRR model, the possible structure of compound **12** remained Glc–GA_52_ (Table S4). We analyzed the potential fragmentation pathways of Glc–GA_52_–DMED (Figure S24), which matched the characteristics of the characteristic fragmentation behaviors of compound **12**. Therefore, we speculated that compound **12** was Glc–GA_52_.

### 3.6. Possible Locations of Potential Glc–GAs in the Metabolic Pathway of GAs 

**Figure 6:**
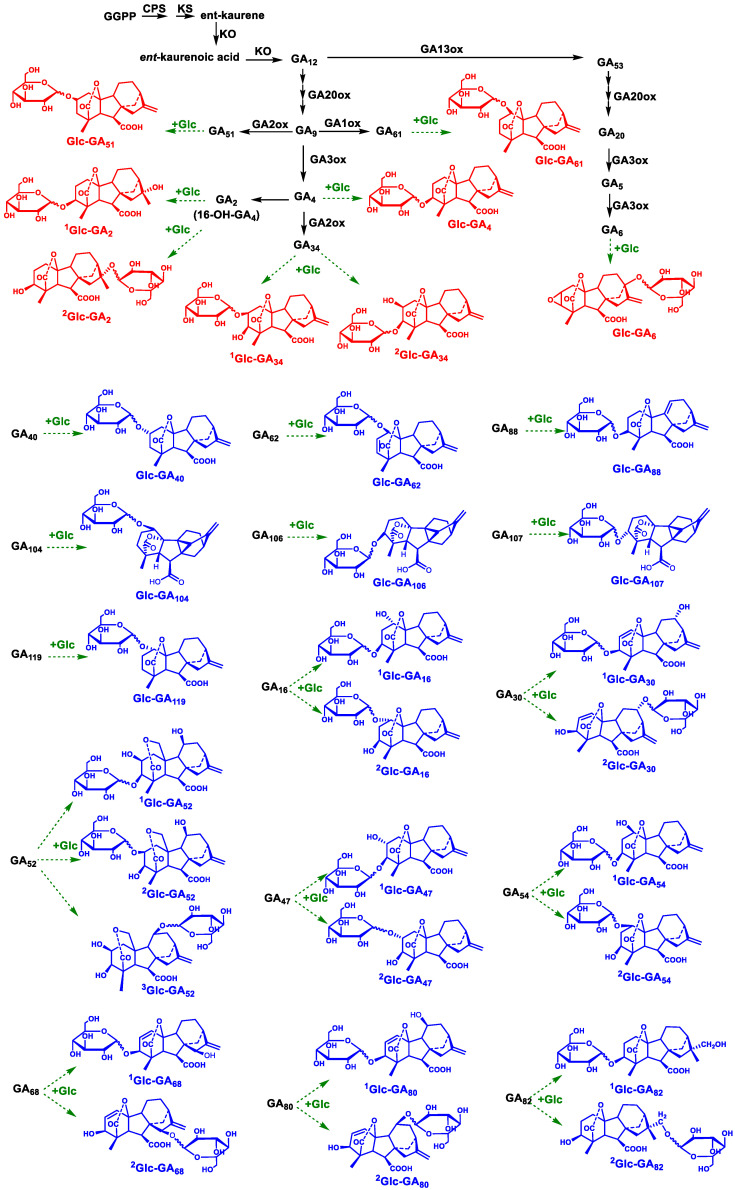
Stereochemical structures of potential Glc–GA compounds and their locations in the GA biosynthetic and inactive pathways. Compounds in red are possible Glc–GAs in a known metabolic pathway. Compounds in blue are Glc–GAs in an unknown metabolic pathway. Solid lines indicate known GA metabolic pathways. Dotted lines indicate possible GA glycosylation processes. The wavy bonds represent unclear anomers. The superscript number in the compound name (Glc–GA_X_) is the corresponding number of possible Glucose–conjugated forms of GA_X_.

One hundred and thirty–six species of gibberellin named after GA_n_ have been reported (Figure S25). In most higher plants, the biosynthesis of GAs typically involves three main steps (Figure 6). First, ent–kaurene is synthesized from trans–geranylgeranyl diphosphate (GGPP) as a precursor. The second step leads to the formation of GA12. Finally, GA12 can be converted into intermediate GAs and bioactive GAs through two pathways. One is the non–13–hydroxylation pathway, where GA12 is directly oxidized to produce intermediate GAs and bioactive GA_4_ and GA_7_. The other pathway is the 13–hydroxylation pathway. GA12 is initially oxidized to form GA53, which is then further oxidized to generate GA_20_, GA_5_, GA_6_, and other intermediate GAs, as well as bioactive GA_1_ and GA_3_. These deactivation and biosynthesis pathways of GAs collectively determine the endogenous content of GAs.

Based on the screening method developed in our study, a total of 12 potential Glc–GAs were screened from six plant samples (Table 1), and their possible structural information is presented in Figure 6. Among these, the positions of the corresponding free–form GAs for the six Glc–GAs (Glc–GA_2_, Glc–GA_4_, Glc–GA_6_, Glc–GA_34_, Glc–GA_51_, and Glc–GA_61_) in the GA metabolic pathway have been clearly elucidated. As shown in Figure 6, within the 13–hydroxylation pathway, intermediates GA_2_, GA_4_, GA_34_, GA_51_, GA_61_, and the bioactive GA_4_ might further undergo glucose conjugation to produce the corresponding glucose conjugated forms (Glc–GA_2_, Glc–GA_6_, Glc–GA_34_, Glc–GA_51_, and Glc–GA_6_). It is noteworthy that we have discovered a new Glucose–conjugated form of GA_4_, GA_4_–3–*O*–glucoside, which has only been reported in previous studies for the glucosyl ester form of GA_4_ (GA_4_ glucosyl ester) and the Glucose–conjugated product of 17–OH–GA_4_ (GA_4_–17–*O*–glucoside). This indicated that GA_4_ may also undergo glucose conjugation at the C–3 hydroxyl position. In addition, the intermediate GA_6_ generated in the non–13–hydroxylation pathway might also undergo glucose conjugation, resulting in Glc–GA_6_. Except for these six Glc–GA species, the positions in the GA metabolic pathway of other Glc–GAs discovered in this study are not clearly reported. Our results might be helpful for studying the biosynthesis and metabolic pathways of these Glc–GAs. These results suggested the widespread occurrence of Glucose–conjugated GAs in plants, where glucose conjugation and the deconjugation of GAs might be involved in regulating the levels of active GAs and play important roles in mediating the conversion between active GAs and intermediate GAs.

## 4. Conclusions

In this study, we successfully developed a structure–guided screening and identification strategy for Glc–GAs. This strategy utilizes characteristic MS/MS fragment ion patterns and incorporates QSRR techniques into the structure identification process. The method offers high sensitivity, requires lower plant sample amounts, and has no bias towards specific activities, enabling the efficient screening and structural identification of low–abundance and low–activity GA compounds. Using this approach, we screened 12 potential Glc–GAs in six plant samples and performed structural elucidation. The established analytical method facilitates the screening of more low–abundance and low–activity or inactive Glc–GAs, as the majority of metabolites in GA metabolic networks are inactive or have low activity. The work is highly meaningful for enhancing our understanding of GA metabolic pathways and investigating the mechanisms of GA functions.

## Figures and Tables

**Table 1:** Summarized information of 12 Glc–GA candidates.

ID	Candidates	Theoretical *m*/*z*		Derivatized Formula	Native Formula	RT	RI	Identification
		DMED –Labeled	*d*_4_–DMED –Labeled					
1	Glc–GA_5, 31, 95, 96, 108, 109, 117, 121, 122,7, 11, 62, 88, 104, 105, 106, 107_	563.2963	567.3214	C_29_H_44_O_9_N_2_	C_25_H_32_O_10_	10.32	718.97	Glc–GA_62, 88, 104,106, 107_^a,b^
2						12.10	760.66	Glc–GA_62, 88, 104,106, 107_^c^
3	Glc–GA_20, 69, 70, 84, 4, 40, 51, 61, 119, 45_	565.3119	569.3370	C_29_H_46_O_9_N_2_	C_25_H_34_O_10_	8.70	682.35	Glc–GA_4, 40, 51, 61, 119_^c^
4						10.79	729.98	Glc–GA_4, 40, 51, 61, 119_^d^
5						13.01	781.97	Glc–GA_4, 40, 51, 61, 119_^a,b,c,e^
6	Glc–GA_126, 3, 6, 30, 80, 92, 94, 22, 68_	579.2912	583.3163	C_29_H_44_O_10_N_2_	C_25_H_32_O_11_	7.47	655.56	Glc–GA_6, 30, 80, 68_^a^
7						8.20	671.46	Glc–GA_6, 30, 80, 68_^e^
8						8.94	687.58	Glc–GA_6, 30_^e^
9						11.12	737.70	Glc–GA_6, 30_^f^
10	Glc–GA_77, 130, 136, 1, 29, 35, 58, 60, 71, 81, 118, 16, 34, 47, 54, 90, 63, 67, 131_	581.3068	585.3320	C_29_H_46_O_10_N_2_	C_25_H_34_O_11_	10.77	729.51	Glc–GA_16, 34, 47, 54_^a^
11	Glc–GA_2, 82_	583.3225	587.3476	C_29_H_48_O_10_N_2_	C_25_H_36_O_11_	8.70	682.35	Glc–GA_2, 82_^e^
12	Glc–GA_125, 129, 23, 52, 13, 17, 46, 66, 99, 102_	611.3174	615.3425	C_30_H_48_O_11_N_2_	C_26_H_36_O_12_	9.55	700.94	Glc–GA_52_^e^

Plant species: ^a^ *Canavalia gladiata*; ^b^ *Phaseolus vulgaris*; ^c^ *Pisum sativum*; ^d^ *Oryza sativa* ssp. *japonica* cv. Nipponbare; ^e^ *Vicia faba*; ^f^ *Vigna angularis*.

## Data Availability

The original contributions presented in the study are included in the article and Supplementary Materials.

## Supplementary Materials

The following supporting information can be downloaded at: https://www.mdpi.com/article/10.3390/metabo14020096/s1, Table S1. Reported Glc–GAs from plant species [28,29,30,31,32,33,34,35,36,37,38,39,40]; Table S2. The exact mass of reported and potential Glc–GAs and the DMED/*d_4_*–DMED derivatization products; Table S3. The screened peak pairs of the proposed *m*/*z* of reported and potential Glc–GA–DMED/*d_4_*–DMED; Table S4. Comparison of the predicted RI of GAs with the experimental RI of Glc–GA candidates; Figure S1. Chemical structures of GA_3_ and Glc–GA_3;_ Figure S2. Total ion chromatogram and extracted ion chromatograms (*m*/*z* 507.1872) of Glc–GA_3_ synthesis reaction products; Figure S3. HR MS/MS spectra of Glc–GA_3_ (*m*/*z* 507.18) at (A) 8.78 min, (B) 9.40 min, and (C) 10.17 min under negative mode; Figure S4. HR MS/MS spectra of compound **1**; Figure S5. HR MS/MS spectra of compound **2**; Figure S6. The proposed fragmentation pathways of Glc–GA_7_–DMED, Glc–GA_62_–DMED, Glc–GA_88_–DMED, Glc–GA_104_–DMED, Glc–GA_105_–DMED, Glc–GA_106_–DMED, and Glc–GA_107_–DMED; Figure S7. Comparison of HRMS/MS spectra of compound **1** and GA_7_; Figure S8. Comparison of HRMS/MS spectra of compound **2** and GA_7_; Figure S9. HR MS/MS spectra of compound **3**; Figure S10. HR MS/MS spectra of compound **4**; Figure S11. HR MS/MS spectra of compound **5**; Figure S12. The proposed fragmentation pathways of Glc–GA_40_–DMED, Glc–GA_51_–DMED, Glc–GA_61_–DMED, Glc–GA_119_–DMED, and Glc–GA_4_–DMED; Figure S13. HR MS/MS spectra of compound **6**; Figure S14. HR MS/MS spectra of compound **7**; Figure S15. HR MS/MS spectra of compound **8**; Figure S16. HR MS/MS spectra of compound **9**; Figure S17. The proposed fragmentation pathways of Glc–GA_92_–DMED, Glc–GA_80_–DMED, Glc–GA_68_–DMED, Glc–GA_3_–DMED, Glc–GA_6_–DMED, and Glc–GA_30_–DMED; Figure S18. HR MS/MS spectra of compound **10**; Figure S19. The proposed fragmentation pathways of Glc–GA_90_–DMED, Glc–GA_16_–DMED, Glc–GA_54_–DMED, Glc–GA_47_–DMED, and Glc–GA_34_–DMED; Figure S20. Comparison of HRMS/MS spectra of compound **10** and GA_34_; Figure S21. HR MS/MS spectra of compound **11**; Figure S22. The proposed fragmentation pathways of Glc–GA_2_–DMED and Glc–GA_82_–DMED; Figure S23. HR MS/MS spectra of compound **12**; Figure S24. The proposed fragmentation pathways of Glc–GA_52_–DMED; Figure S25. The structures of reported GAs.
