# Supplementary material for: Advancing Glucose Conjugated Gibberellins Discovery: A Structure–Oriented Screening and Identification Method for Unraveling Gibberellin Metabolites in Plants
Source: Metabolites. 2024 Jan 29;14(2):96. doi: 10.3390/metabo14020096 (PMC10890662; doi:10.3390/metabo14020096)
Supplement: Supplementary file 1 [file metabolites-14-00096-s001.zip › metabolites-2814973-supplementary.pdf]

Supplementary Materials

List of tables and figures

Table S1. Reported Glc-GAs from plant species.

Table S2. The exact mass of reported and potential Glc-GAs and the DMED/d_4-DMED derivatization products.

Table S3. The screened peak pairs of the proposed *m/z* of reported and potential Glc-GAs-DMED/d_4-DMED.

Table S4. Comparison of the predicted RI of GAs with the experimental RI of Glc-GAs candidates.

Figure S1. Chemical structures of GA_3 and Glc-GA_3.

Figure S2. Total ion chromatogram and extracted ion chromatograms (*m/z* 507.1872) of Glc-GA_3 synthesis reaction products.

Figure S3. HR MS/MS spectras of Glc-GA_3 (*m/z* 507.18) at (A)8.78 min, (B)9.40 min, (C)10.17 min under negative mode.

Figure S4. HR MS/MS spectra of compound 1.

Figure S5. HR MS/MS spectra of compound 2.

Figure S6. The proposed fragmentation pathways of Glc-GA_7-DMED, Glc-GA_{62}-DMED, Glc-GA_{88}-DMED, Glc-GA_{104}-DMED, Glc-GA_{105}-DMED, Glc-GA_{106}-DMED and Glc-GA_{107}-DMED.

Figure S7. Comparison of HRMS/MS spectra of compound 1 and GA_7.

Figure S8. Comparison of HRMS/MS spectra of compound 2 and GA_7.

Figure S9. HR MS/MS spectra of compound 3.

Figure S10. HR MS/MS spectra of compound 4.

Figure S11. HR MS/MS spectra of compound 5.

Figure S12. The proposed fragmentation pathways of Glc-GA_{40}-DMED, Glc-GA_{51}-DMED, Glc-GA_{61}-DMED, Glc-GA_{119}-DMED and Glc-GA_4-DMED.

Figure S13. HR MS/MS spectra of compound 6.

Figure S14. HR MS/MS spectra of compound 7.

Figure S15. HR MS/MS spectra of compound 8.

Figure S16. HR MS/MS spectra of compound 9.

Figure S17. The proposed fragmentation pathways of Glc-GA_{92}-DMED, Glc-GA_{80}-DMED, Glc-GA_{68}-DMED, Glc-GA_{3}-DMED, Glc-GA_{6}-DMED and Glc-GA_{30}-DMED.

Figure S18. HR MS/MS spectra of compound 10.

Figure S19. The proposed fragmentation pathways of Glc-GA_{90}-DMED, Glc-GA_{16}-DMED, Glc-GA_{54}-DMED, Glc-GA_{47}-DMED and Glc-GA_{34}-DMED.

Figure S20. Comparison of HRMS/MS spectra of compound 10 and GA_{34}.

Figure S21. HR MS/MS spectra of compound 11.

Figure S22. The proposed fragmentation pathways of Glc-GA_{2}-DMED and Glc-GA_{82}-DMED.

Figure S23. HR MS/MS spectra of compound 12.

Figure S24. The proposed fragmentation pathways of Glc-GA_{52}-DMED.

Figure S25. The structures of reported GAs.

Table S1. Reported Glc-GAs from plant species.

Glc-GAs	Structures	Formula	Sample species	Detection methods	References
GA1-3-*O*-glucoside	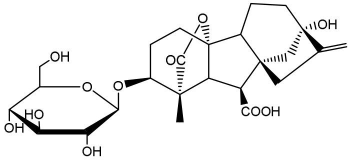	$C_{25}H_{34}O_{11}$	Carrot,<br>Anise somatic,<br>Dalbergia dolichopetala,<br>Phaseolus coccineus,<br>Maize caryopsis	HPLC-RC，<br>GLC-RC，<br>GC-MS	[17,21,28]
GA1-13-*O*-glucoside	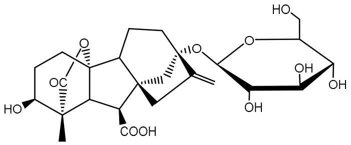	$C_{25}H_{34}O_{11}$	Anise somatic,<br>Phaseolus coccineus,<br>Maize caryopsis	HPLC-RC，<br>GLC-RC，<br>GC-MS	[21,29,30]
GA3-3-*O*-glucoside	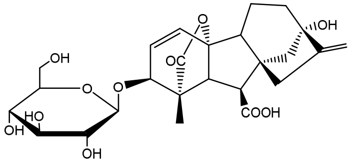	$C_{25}H_{32}O_{11}$	Pharbitis nil,<br>Phaseolus coccineus,<br>Dalbergia dolichopetala,<br>Quamoclit pennata	HPLC，<br>GC-MS	[17-18,31-32]
16,17-dihydro-16,17-dihydroxy-GA4-17-*O*-glucoside	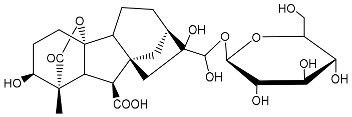	$C_{25}H_{36}O_{13}$	Anise somatic,<br>Dalbergia dolichopetala	HPLC-RC，<br>GLC-RC，<br>GC-MS	[17]

16α,17-dihydroxy-16,17-dihydro-GA4-17-*O*-β-D-glucoside	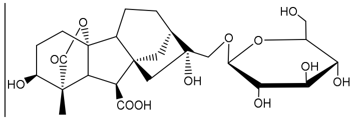	$C_{25}H_{36}O_{12}$	Rice anthers	HPLC	[33]
GA5-13-*O*-glucoside	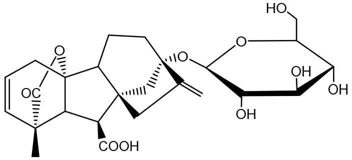	$C_{25}H_{32}O_{10}$	Phaseolus coccineus,<br>Maize caryopsis	HPLC，<br>GC-MS	[21,29]
GA8-2-*O*-glucoside	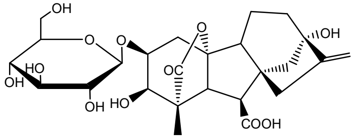	$C_{25}H_{34}O_{12}$	Phaseolus vulgaris,<br>Anise somatic,<br>Pharbitis nil,<br>Phaseolus coccineus,<br>Dalbergia dolichopetala,<br>Maize seedlings,<br>Althaea rosea	HPLC-RC，<br>GC-MS	[5,18,28,34]
GA20-13-*O*-glucoside	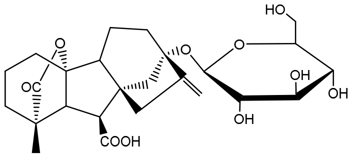	$C_{25}H_{34}O_{10}$	Maize seedlings,<br>Pisum sativum,<br>Barley varietie	HPLC，<br>GC-MS	[20,22]
GA26-2-*O*-glucoside	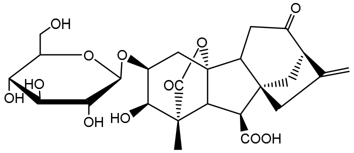	$C_{25}H_{32}O_{12}$	Pharbitis nil	NMR,<br>GC-MS	[39]

GA_{27}-2-*O*-glucoside	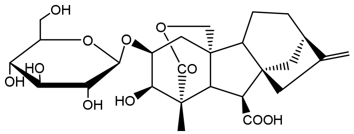	$C_{26}H_{36}O_{11}$	Pharbitis nil	NMR, GC-MS	[35]
GA_{29}-2-*O*-glucoside	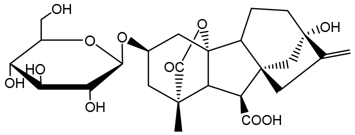	$C_{25}H_{34}O_{11}$	Pharbitis nil, Phaseolus coccineus, Pisum sativum, Maize seedlings	HPLC， GC-MS	[20,22,29,36]
GA_{29}-13-*O*-glucoside	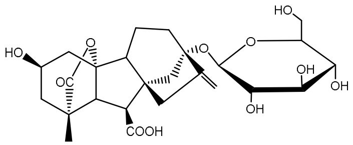	$C_{25}H_{34}O_{11}$	Phaseolus coccineus, Pisum sativum	LC-ESI-tandem-MS， GC-MS	[20,30]
GA_{35}-11-*O*-glucoside	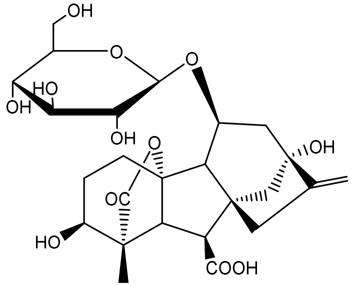	$C_{25}H_{34}O_{12}$	Phaseolus coccineus, Cytisus scoparius	NMR， GC-MS	[18,37]

GA_1 glucosyl ester	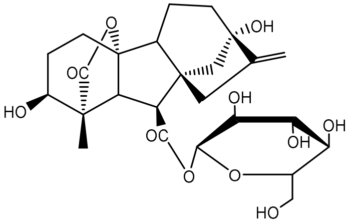	$C_{25}H_{34}O_{11}$	Phaseolus vulgaris,<br>Anise somatic,<br>Phaseolus coccineus	NMR, TLC，<br>HPLC-RC，<br>GC-MS	[18,33]
GA_4 glucosyl ester	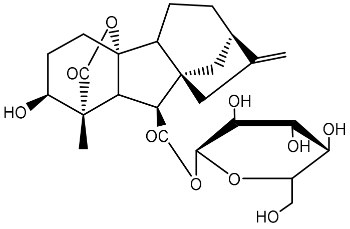	$C_{25}H_{34}O_{10}$	Phaseolus vulgaris,<br>Anise somatic,<br>Phaseolus coccineus	TLC，<br>GC-MS	[18,33,38]
GA_5 glucosyl ester	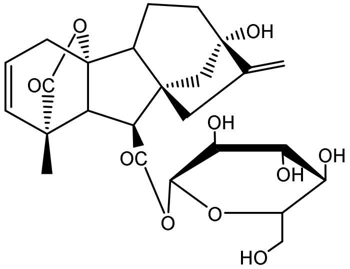	$C_{25}H_{32}O_{10}$	Pharbitis purpurea	HPLC，<br>GC-MS	[38]
GA_9 glucosyl ester	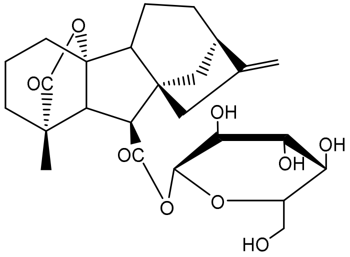	$C_{25}H_{34}O_9$	Picea sitthensis,<br>Sitka spruce	GC-MS，<br>HPLC-MS	[23,40]

GA_{37} glucosyl ester	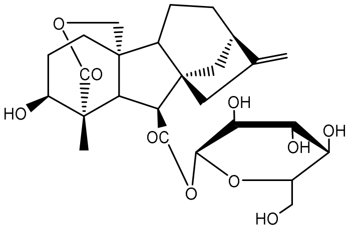	$C_{26}H_{36}O_{10}$	Phaseolus vulgaris, Pharbitis purpurea	TLC, HPLC, GC-MS	[34,38-39]
GA_{38} glucosyl ester	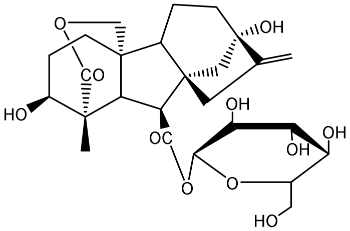	$C_{26}H_{36}O_{11}$	Phaseolus vulgaris	TLC, GC-MS	[38]
GA_{44} glucosyl ester	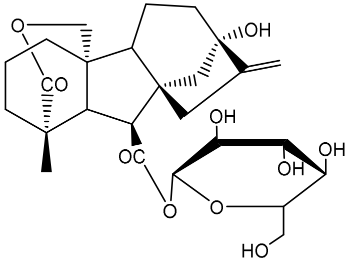	$C_{26}H_{36}O_{10}$	Pharbitis purpurea	HPLC, GC-MS	[39]

Table S2. The exact mass of reported and potential Glc-GAs and the DMED/d_4-DMED derivatization products.

Glucose conjugated GA_n（Glc-GA_n）	*m/z*	
	Glc-GA_n	
	DMED-labeled	d_4-DMED-labeled
Glc-$GA_{73, 103, 120}$	547.3014	551.3265
Glc-GA_9	549.3170	553.3421
Glc-GA5$_{, 7, 11,\ 31, 62, 88, 95, 96, 104, 105, 106, 107, 108, 109, 117, 121, 122}$	563.2963	567.3214
Glc-GA_{15}	563.3327	567.3578
Glc-$GA_{4, 20, 40, 45, 51, 61, 69, 70, 84, 119}$	565.3119	569.3370
Glc-GA_{12}	565.3483	569.3734
Glc-GA_{10}	567.3275	571.3526
Glc-$GA_{3, 6, 22, 30, 68, 80, 92, 94, 126}$	579.2912	583.3163
Glc-$GA_{24, 37, 44, 64, 113, 114, 134}$	579.3276	583.3527
Glc-$GA_{1, 16, 29, 34, 35, 47, 54, 58, 60, 63, 67, 71, 77, 81, 90, 118, 130, 131, 136}$	581.3068	585.3320
Glc-$GA_{14, 53, 110, 111, 112, 133}$	581.3432	585.3683
Glc-$GA_{2,\ 82}$	583.3225	587.3476
Glc-GA_{59}	593.2705	597.2956
Glc-$GA_{21, 26, 33, 87, 93,}$	595.2861	599.3112
Glc-$GA_{19, 25, 27, 36, 38, 65, 98, 101, 115, 116, 124, 128}$	595.3225	599.3476
Glc-$GA_{8, 48, 49, 50, 55, 56,57, 72, 76, 78,\ 79, 85, 91, 132}$	597.3018	601.3269
Glc-$GA_{18, 74, 97, 100, 123, 127, 135}$	597.3381	601.3633
Glc-$GA_{42,\ 83}$	599.3538	603.3789
Glc-GA_{32}	611.2810	615.3061
Glc-$GA_{13, 17, 23, 46, 52, 66, 99, 102, 125, 129}$	611.3174	615.3425
Glc-$GA_{75, 86}$	613.2967	617.3218
Glc-$GA_{28, 39, 43}$	627.3123	631.3374
Glc-GA_{89}	629.2916	633.3167
Glc-GA_{41}	629.3280	633.3531

Table S3. The screened peak pairs of the proposed *m/z* of reported and potential Glc-GAs-DMED/d_4-DMED.

N.	DMED-labeled			d_4-DMED-labeled		
	m/z	RT（min）	Intensity	*m/z*	RT（min）	Intensity
			Canavalia gladiata			
1	563.2858	13.14	2.57E+04	567.3103	13.04	1.65E+04
2	563.2864	7.14	5.18E+04	567.3116	7.08	3.66E+04
3	563.2853	5.62	4.51E+04	567.3111	5.45	3.28E+04
4	563.2962	9.88	1.52E+05	567.3214	9.92	7.84E+04

5	563.2962	10.32	5.84E+05	567.3218	10.33	6.66E+05
6	563.2963	12.34	2.22E+04	567.3216	12.30	1.41E+04
7	563.2963	15.14	5.47E+04	567.3216	15.21	5.91E+04
8	563.2963	19.16	5.69E+04	567.3216	19.28	5.88E+04
9	563.2963	19.68	7.53E+04	567.3216	19.55	5.91E+04
10	563.2963	19.28	1.53E+05	567.3216	19.38	1.52E+05
11	565.3117	9.87	1.09E+05	569.3372	9.86	5.77E+04
12	565.3114	13.00	5.90E+05	569.3373	13.05	5.12E+05
13	579.2911	7.48	6.45E+05	583.3161	7.47	6.76E+05
14	579.2911	13.76	8.68E+04	583.3161	13.96	4.46E+04
15	579.2911	15.82	5.01E+05	583.3161	15.81	6.00E+04
16	579.2911	16.34	3.61E+05	583.3161	16.47	6.76E+04
17	581.3026	8.45	1.74E+05	585.3315	8.35	3.17E+05
18	581.3054	14.73	2.80E+05	585.3315	14.64	3.75E+05
19	581.3055	20.18	2.56E+04	585.3315	20.23	3.09E+04
20	581.3065	13.07	2.96E+04	585.3317	12.98	4.20E+04
21	581.3065	13.26	1.06E+05	585.3317	12.95	7.82E+05
22	581.3067	13.29	4.77E+05	585.3319	13.20	7.70E+05
23	581.3068	10.77	1.46E+05	585.3320	10.80	7.70E+05
24	581.3068	13.81	2.54E+05	585.3320	13.92	3.51E+05
25	581.3068	16.09	6.67E+05	585.3320	15.98	7.26E+05
Phaseolus vulgaris						
26	563.2902	15.70	5.04E+04	567.3145	15.71	6.94E+04
27	563.2902	15.62	2.67E+05	567.3145	15.61	1.57E+05
28	563.2902	9.90	2.67E+05	567.3152	9.90	1.55E+05
29	563.2962	3.66	5.84E+05	567.3218	3.57	6.66E+05
30	563.2962	4.39	4.44E+04	567.3218	4.24	4.57E+04
31	563.2962	5.78	1.98E+05	567.3218	5.62	2.18E+05
32	563.2962	10.31	4.84E+05	567.3218	10.33	5.23E+05
33	563.3165	5.78	1.98E+05	567.3411	5.62	2.18E+05
34	563.3168	4.39	4.44E+04	567.3414	4.24	4.57E+04
35	565.3032	9.36	4.50E+04	569.2280	9.17	5.15E+04
36	565.3033	15.89	5.98E+04	569.3280	15.88	3.41E+04
37	565.3118	12.99	1.22E+05	569.3396	12.99	1.14E+05
38	565.3118	16.84	3.28E+04	569.3396	16.84	2.06E+04
39	565.3120	18.67	2.53E+04	569.3395	18.48	4.27E+04
Pisum sativum						
40	563.2963	3.66	5.84E+05	567.3218	3.57	6.66E+05
41	563.2963	10.31	1.46E+05	567.3218	10.42	7.70E+04
42	563.2963	12.09	9.95E+05	567.3218	12.15	8.48E+05
43	565.3117	7.48	2.45E+05	569.3390	7.47	1.76E+05
44	565.3118	8.70	2.27E+05	569.3396	8.65	1.25E+05
45	565.3118	9.96	2.13E+04	569.3396	9.89	1.42E+04

46	565.3118	9.96	2.13E+04	569.3396	10.13	2.44E+04
47	565.3118	12.02	1.53E+05	569.3396	11.96	1.52E+05
48	565.3118	12.98	1.09E+05	569.3396	13.05	5.77E+04
49	565.3118	13.62	5.90E+05	569.3396	13.85	5.12E+05
50	565.3118	20.46	4.33E+04	569.3396	20.34	3.97E+04
51	565.3119	24.07	2.15E+04	569.3396	23.89	1.76E+04
Oryza sativa ssp. japonica cv. Nipponbare						
52	565.3117	7.48	2.45E+05	569.3390	7.47	1.76E+05
53	565.3118	8.70	2.27E+05	569.3396	8.65	1.25E+05
54	565.3118	8.96	2.13E+04	569.3396	8.89	1.42E+04
55	565.3118	8.96	2.13E+04	569.3396	9.13	2.44E+04
56	565.3118	9.02	1.53E+05	569.3396	8.96	1.52E+05
57	565.3118	9.98	1.09E+05	569.3396	10.05	5.77E+04
58	565.3118	10.79	1.59E+06	569.3396	10.84	1.51E+06
Vicia faba						
59	565.3117	9.87	1.09E+05	569.3390	9.86	5.77E+04
60	565.3117	10.64	1.68E+06	569.3396	10.62	8.48E+05
61	565.3118	10.73	2.17E+05	569.3396	10.67	3.96E+05
62	565.3118	11.63	1.81E+05	569.3396	11.66	1.39E+05
63	565.3118	12.34	2.01E+05	569.3397	12.17	1.36E+05
64	565.3119	13.01	5.90E+05	569.3396	13.09	5.12E+05
65	565.3119	17.15	1.42E+05	569.3396	17.24	8.13E+04
66	579.2911	3.80	1.45E+04	583.3161	3.69	1.49E+04
67	579.2911	7.74	9.07E+04	583.3161	7.91	6.37E+04
68	579.2911	8.20	7.32E+04	583.3161	8.19	4.87E+04
69	579.2911	9.57	3.84E+05	583.3161	9.56	2.26E+05
70	579.2912	5.53	3.33E+04	583.3161	5.43	5.55E+04
71	579.2912	6.34	9.66E+04	583.3162	6.33	8.18E+04
72	579.2912	8.20	1.68E+06	583.3162	8.32	8.48E+05
73	579.2912	8.94	1.81E+06	583.3162	8.88	1.32E+06
74	579.2912	11.85	5.07E+04	583.3162	12.03	6.68E+04
75	579.2912	23.71	1.28E+04	583.3162	23.90	1.90E+04
76	579.2913	12.34	2.01E+05	583.3163	12.17	1.36E+05
77	579.2913	13.43	3.57E+04	583.3163	13.28	2.89E+04
78	583.3224	9.96	2.13E+04	587.3475	9.89	1.42E+04
79	583.3224	9.96	2.13E+04	587.3475	10.13	2.44E+04
80	583.3224	10.14	5.40E+04	587.3475	10.06	7.06E+04
81	583.3225	10.77	8.50E+05	587.3476	10.65	6.60E+05
82	583.3225	16.34	3.61E+04	587.3476	16.47	6.76E+04
83	611.3171	3.90	1.04E+05	615.3423	3.87	7.12E+04
84	611.3171	4.97	2.60E+05	615.3424	4.96	1.74E+05
85	611.3174	7.74	9.07E+04	615.3425	7.91	6.37E+04
86	611.3174	8.20	7.32E+04	615.3425	8.19	4.87E+04

87	611.3174	9.55	7.45E+05	615.3425	9.53	5.94E+05
			Vigna angularis			
88	579.2914	10.51	4.52E+04	583.3165	10.49	4.32E+04
89	579.2914	11.12	5.65E+05	583.3165	11.07	5.73E+05
90	579.2915	12.25	1.12E+04	583.3174	12.23	1.04E+04
91	579.2915	12.46	9.95E+05	583.3174	12.46	8.48E+05
92	579.2915	14.74	4.08E+04	583.3174	14.72	8.11E+04
93	579.2915	17.15	1.42E+05	583.3174	17.24	8.13E+04

Table S4. Comparison of the predicted RI of GAs with the experimental RI of Glc-GAs candidates.

Co.ID	$RI_{Glc\text{-}GAs}$ (experimental)	Candidates	RI_{GAs} (predicted)	Difference
1	718.97	Glc-GA_{105}	535.52	-183.45
		Glc-GA_{62}	685.27	-33.70
		Glc-GA_{104}	716.94	-2.03
		Glc-GA_{106}	796.48	77.51
		Glc-GA_{107}	826.57	107.60
		Glc-GA_{7}	840.94	121.97
		Glc-GA_{88}	865.36	146.39
Co.ID	$RI_{Glc\text{-}GAs}$ (experimental)	Candidates	RI_{GAs} (predicted)	Difference
2	760.66	Glc-GA_{105}	535.52	-225.14
		Glc-GA_{62}	685.27	-75.39
		Glc-GA_{104}	716.94	-43.72
		Glc-GA_{106}	796.48	35.82
		Glc-GA_{107}	826.57	65.91
		Glc-GA_{7}	840.94	80.28
		Glc-GA_{88}	865.36	104.70
Co.ID	$RI_{Glc\text{-}GAs}$ (experimental)	Candidates	RI_{GAs} (predicted)	Difference
3	682.35	Glc-GA_{40}	742.08	59.73
		Glc-GA_{61}	767.97	85.62
		Glc-GA_{51}	775.88	93.53
		Glc-GA_{119}	827.14	144.79
		Glc-GA_{4}	844.67	162.32
Co.ID	$RI_{Glc\text{-}GAs}$ (experimental)	Candidates	RI_{GAs} (predicted)	Difference
4	729.98	Glc-GA_{40}	742.08	12.10
		Glc-GA_{61}	767.97	37.99
		Glc-GA_{51}	775.88	45.90

		Candidates		
		Glc-GA119	827.14	97.16
		Glc-GA4	844.67	114.69

Co.ID	RIGlc-GAs (experimental)	Candidates	RIGAs (predicted)	Difference
5	781.97	Glc-GA40	742.08	-39.89
		Glc-GA61	767.97	-14.00
		Glc-GA51	775.88	-6.09
		Glc-GA119	827.14	45.17
		Glc-GA4	844.67	62.70

Co.ID	RIGlc-GAs (experimental)	Candidates	RIGAs (predicted)	Difference
6	655.56	Glc-GA92	578.58	-76.98
		Glc-GA80	605.53	-50.03
		Glc-GA68	614.72	-40.84
		Glc-GA3	663.43	7.87
		Glc-GA6	688.29	32.73
		Glc-GA30	813.38	157.82

Co.ID	RIGlc-GAs (experimental)	Candidates	RIGAs (predicted)	Difference
7	671.46	Glc-GA92	578.58	-92.88
		Glc-GA80	605.53	-65.93
		Glc-GA68	614.72	-56.74
		Glc-GA3	663.43	-8.03
		Glc-GA6	688.29	16.83
		Glc-GA30	813.38	141.92

Co.ID	RIGlc-GAs (experimental)	Candidates	RIGAs (predicted)	Difference
8	687.58	Glc-GA92	578.58	-109.00
		Glc-GA80	605.53	-82.05
		Glc-GA68	614.72	-72.86
		Glc-GA3	663.43	-24.15
		Glc-GA6	688.29	0.71
		Glc-GA30	813.38	125.80

Co.ID	RIGlc-GAs (experimental)	Candidates	RIGAs (predicted)	Difference
9	737.70	Glc-GA92	578.58	-159.12
		Glc-GA80	605.53	-132.17
		Glc-GA68	614.72	-122.98
		Glc-GA3	663.43	-74.27
		Glc-GA6	688.29	-49.41
		Glc-GA30	813.38	75.68

Co.ID	RIGlc-GAs (experimental)	Candidates	RIGAs (predicted)	Difference
10	729.51	Glc-GA90	646.06	-83.45

		Glc-GA$_{54}$	680.55	-48.96
		Glc-GA$_{47}$	722.13	-7.38
		Glc-GA$_{34}$	763.45	33.94
		Glc-GA$_{16}$	812.01	82.50
Co.ID	$RI_{Glc-GAs}$ (experimental)	Candidates	RI_{GAs} (predicted)	Difference
11	682.35	Glc-GA$_{82}$	625.33	-57.02
		Glc-GA$_{2}$	654.94	-27.41
Co.ID	$RI_{Glc-GAs}$ (experimental)	Candidates	RI_{GAs} (predicted)	Difference
12	700.94	Glc-GA$_{52}$	663.11	-37.83

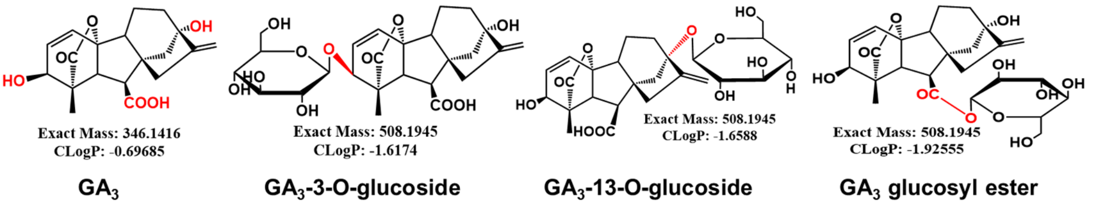

Figure S1. Chemical structures of GA_3 and Glc-GA_3.

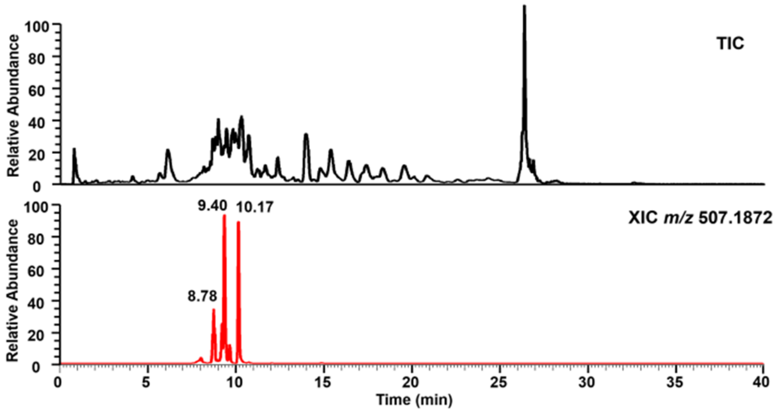

Figure S2. Total ion chromatogram and extracted ion chromatograms (*m/z* 507.1872) of Glc-GA_3 synthesis reaction products.

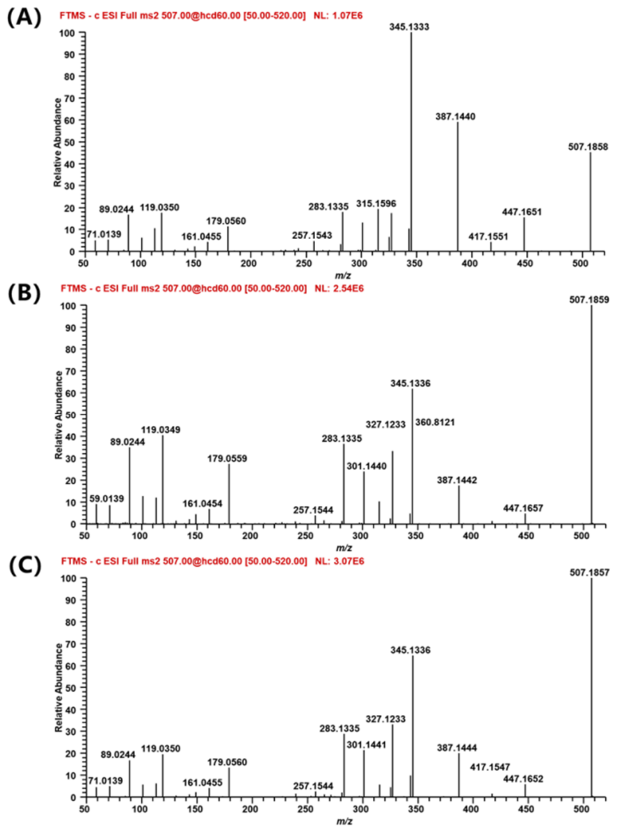

Figure S3. HR MS/MS spectras of Glc-GA_3 (*m/z* 507.18) at (A)8.78 min, (B)9.40 min, (C)10.17 min under negative mode.

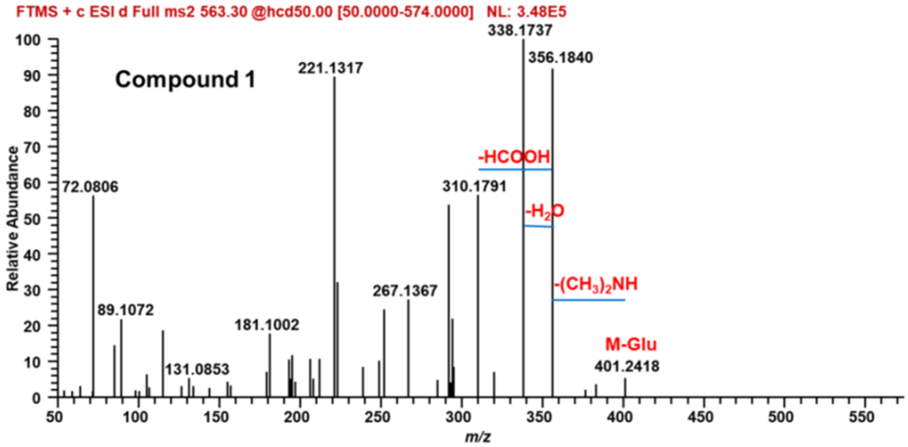

Figure S4. HR MS/MS spectra of compound 1 under positive mode.

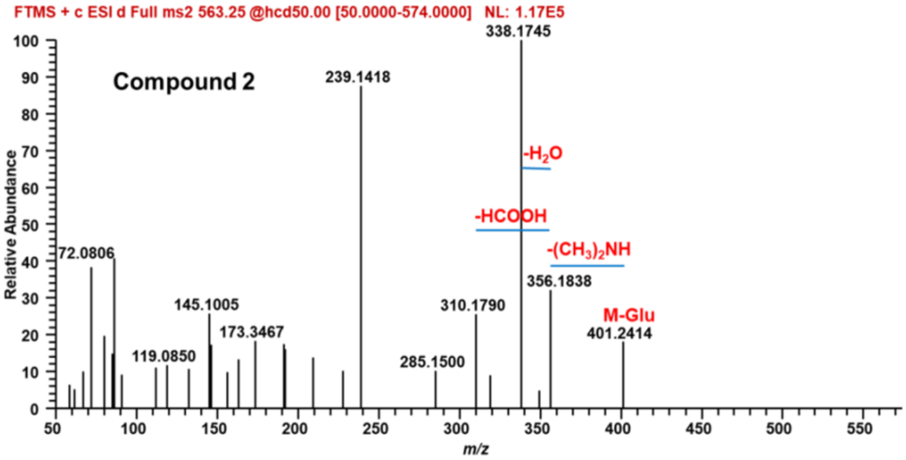

Figure S5. HR MS/MS spectra of compound 2 under positive mode.

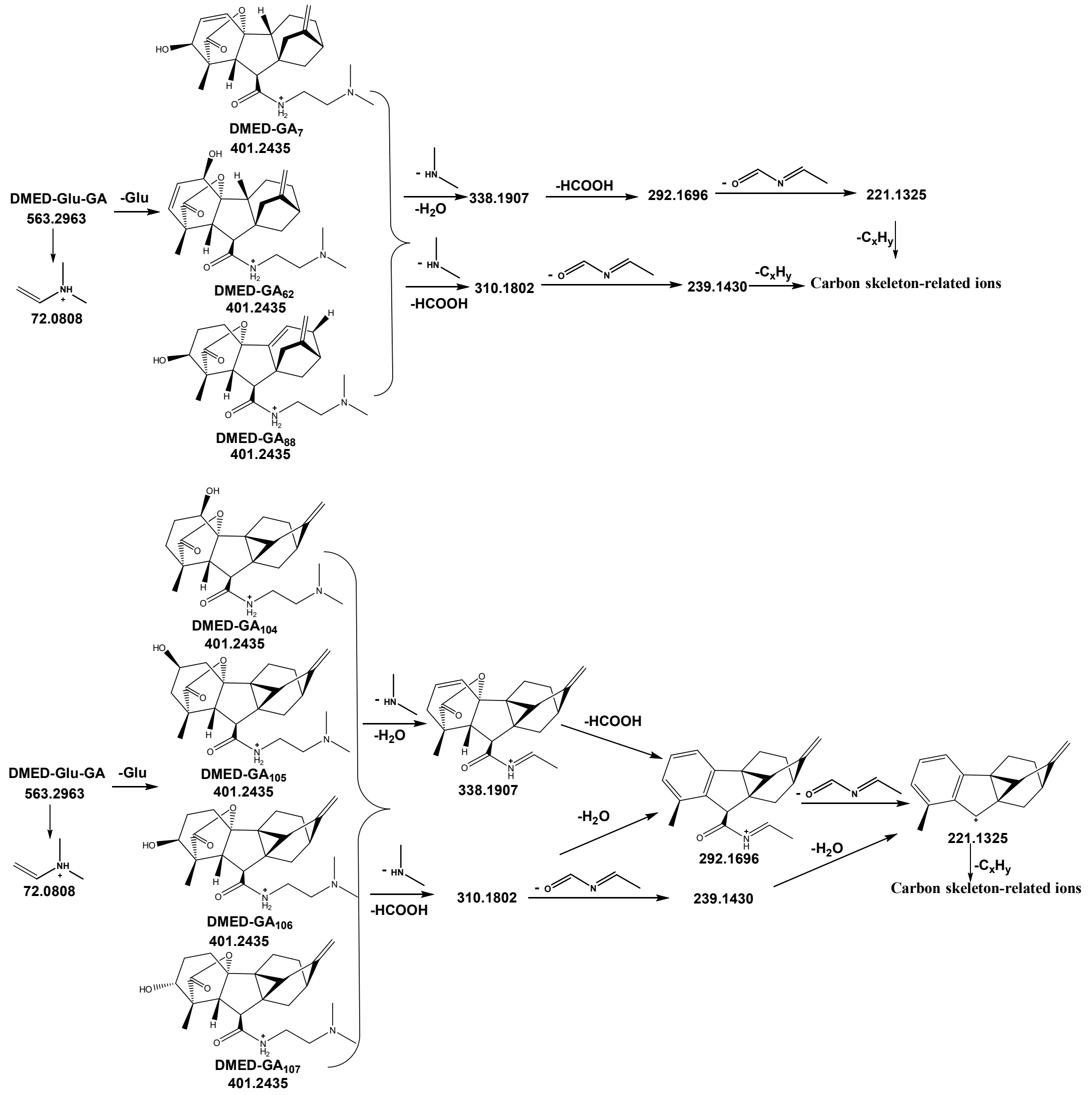

Figure S6. The proposed fragmentation pathways of Glc-GA7-DMED, Glc-GA62-DMED, Glc-GA88-DMED, Glc-GA104-DMED, Glc-GA105-DMED, Glc-GA106-DMED and

Glc-GA107-DMED.

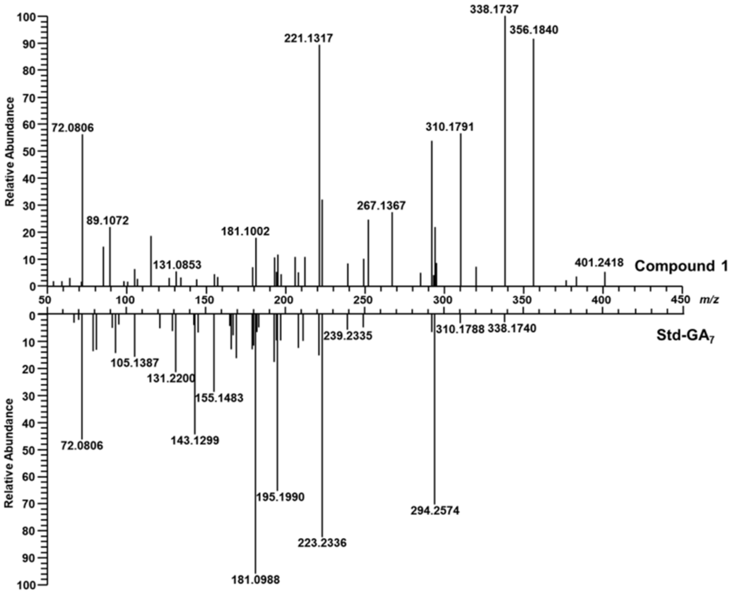

Figure S7. Comparison of HRMS/MS spectra of compound 1 and GA_7.

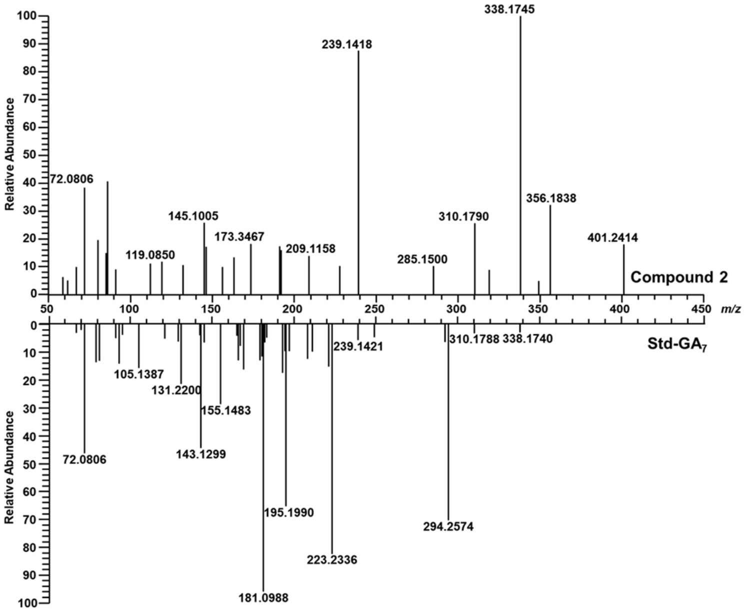

Figure S8. Comparison of HRMS/MS spectra of compound 2 and GA_7.

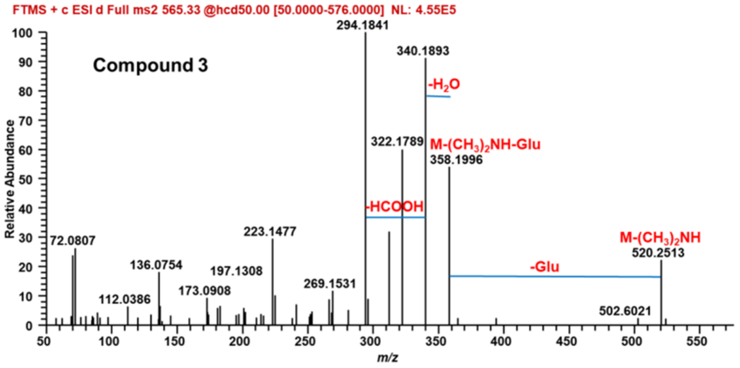

Figure S9. HR MS/MS spectra of compound 3 under positive mode.

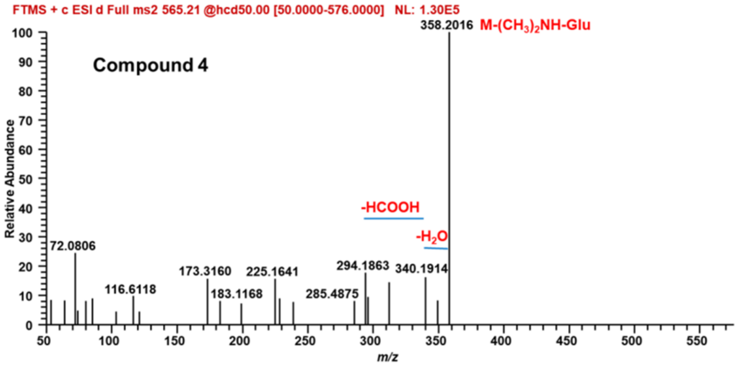

Figure S10. HR MS/MS spectra of compound 4 under positive mode.

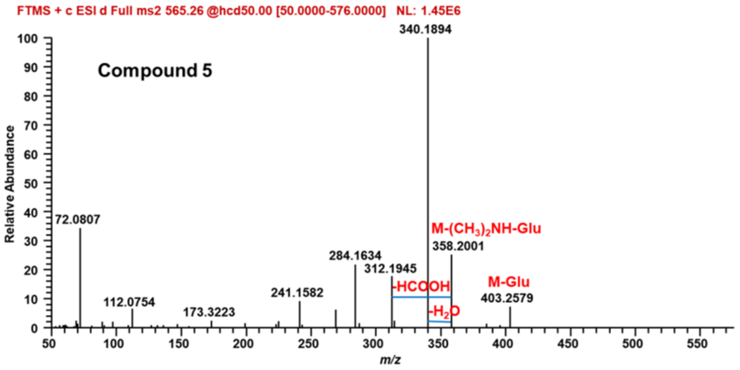

Figure S11. HR MS/MS spectra of compound 5 under positive mode.

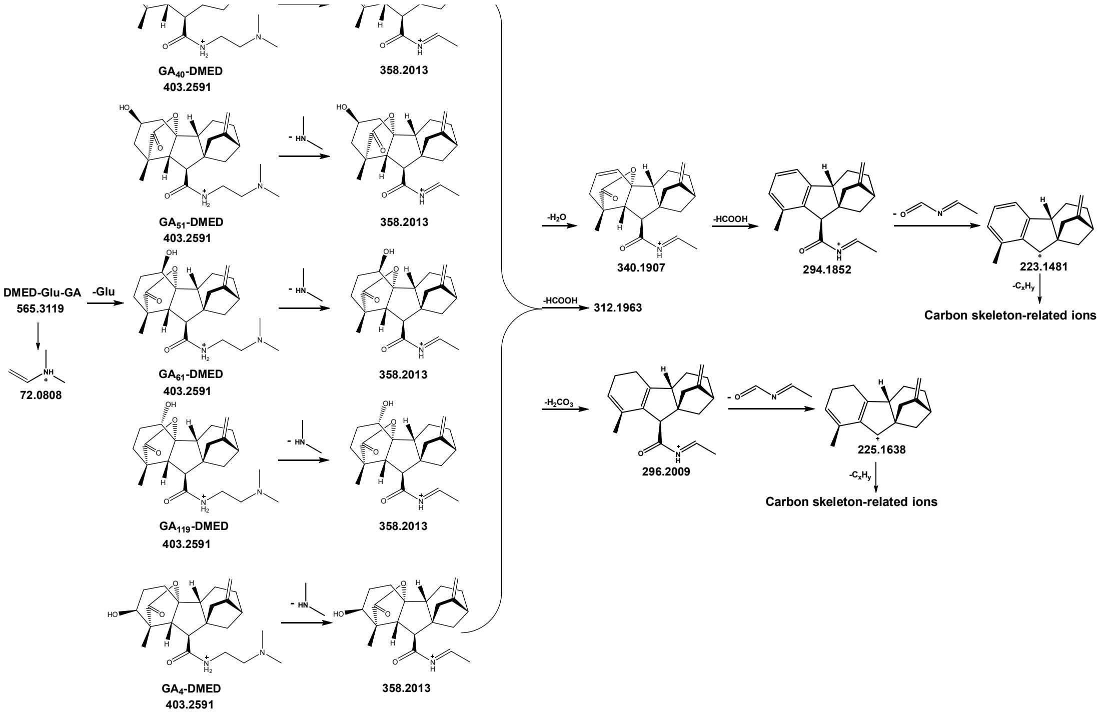

Figure S12. The proposed fragmentation pathways of Glc-GA40-DMED, Glc-GA51-DMED, Glc-GA61-DMED, Glc-GA119-DMED and Glc-GA4-DMED.

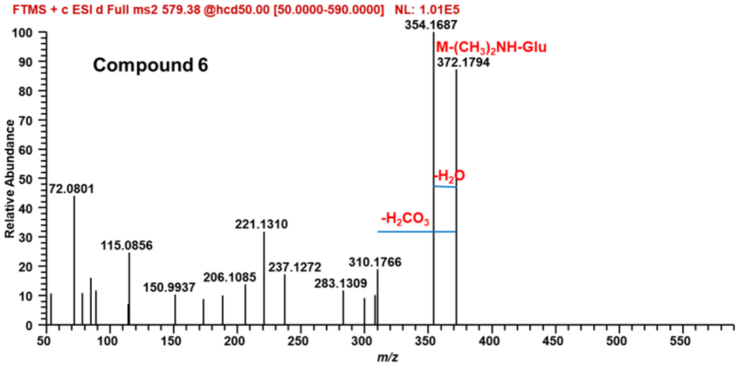

Figure S13. HR MS/MS spectra of compound 6 under positive mode.

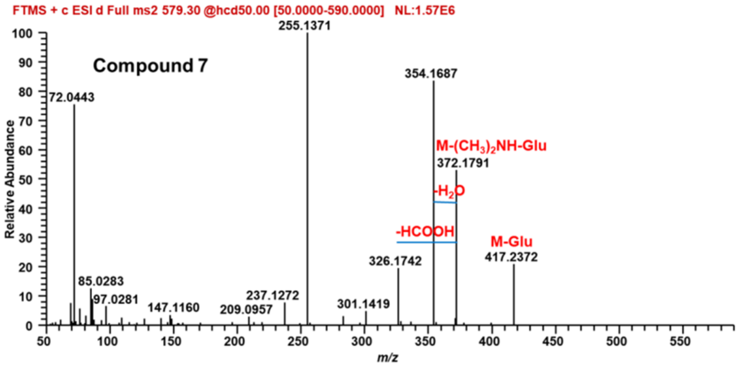

Figure S14. HR MS/MS spectra of compound 7 under positive mode.

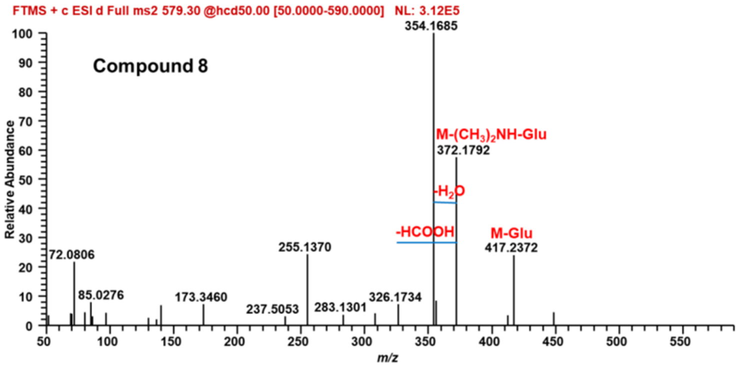

Figure S15. HR MS/MS spectra of compound 8 under positive mode.

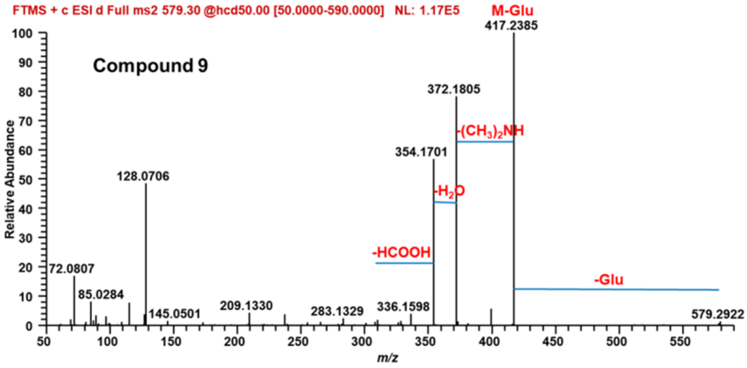

Figure S16. HR MS/MS spectra of compound 9 under positive mode.

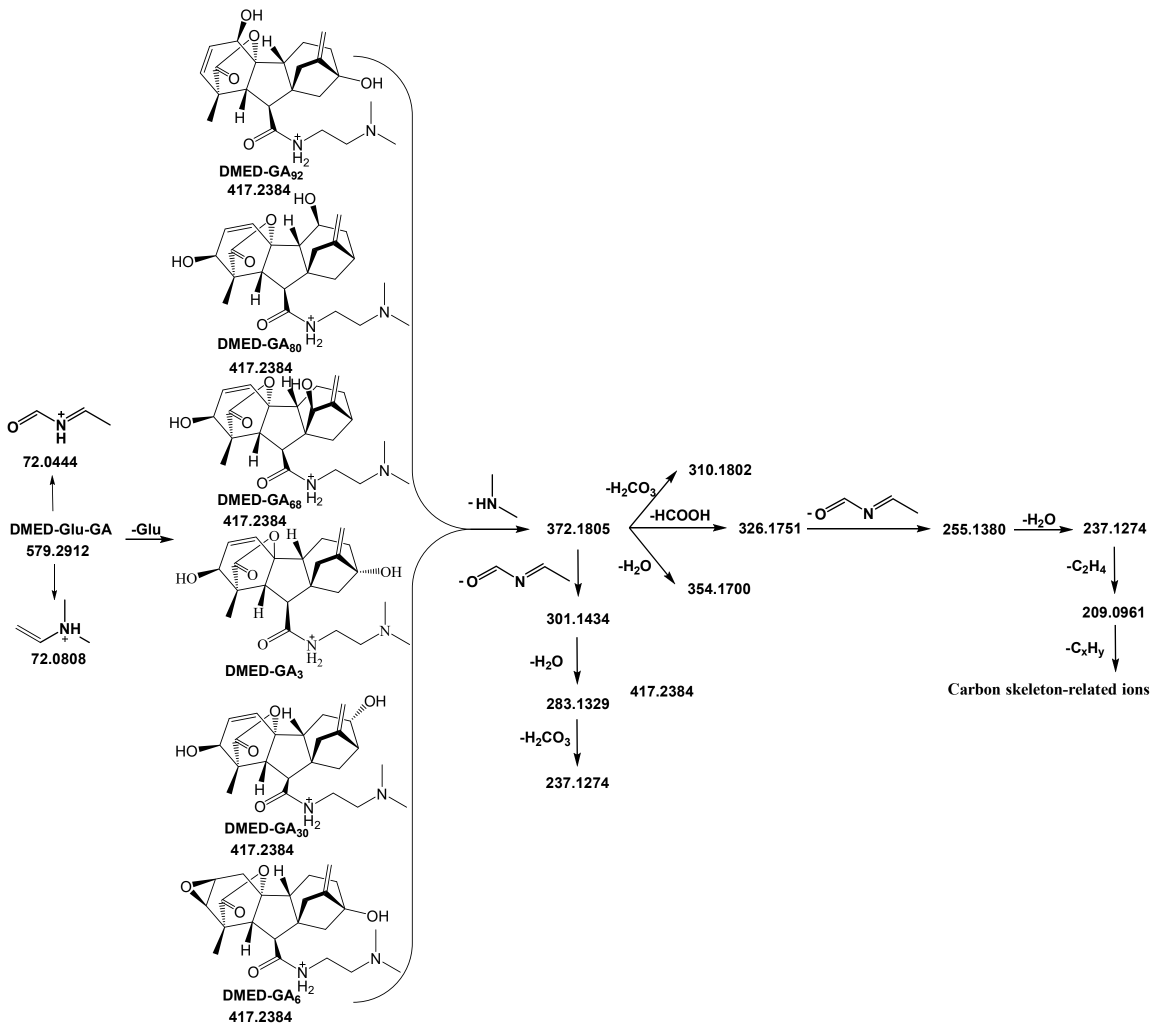

Figure S17. The proposed fragmentation pathways of Glc-GA92-DMED, Glc-GA80-DMED, Glc-GA68-DMED, Glc-GA3-DMED, Glc-GA6-DMED and Glc-GA30-DMED.

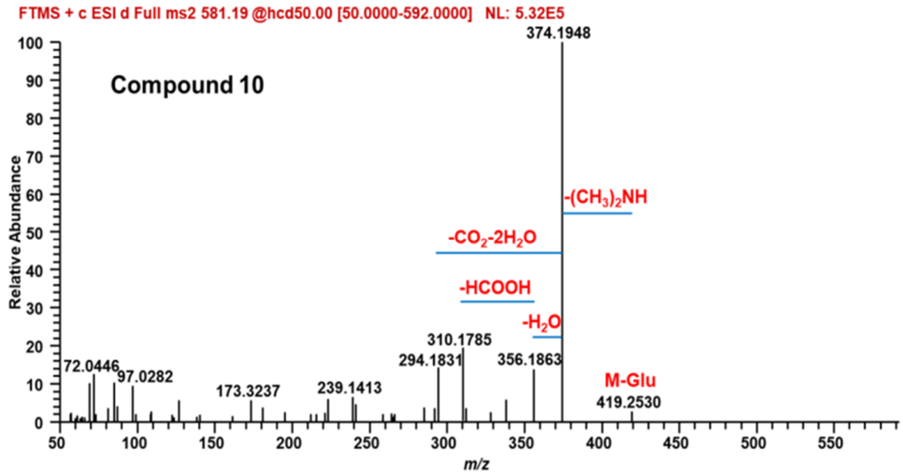

Figure S18. HR MS/MS spectra of compound 10 under positive mode.

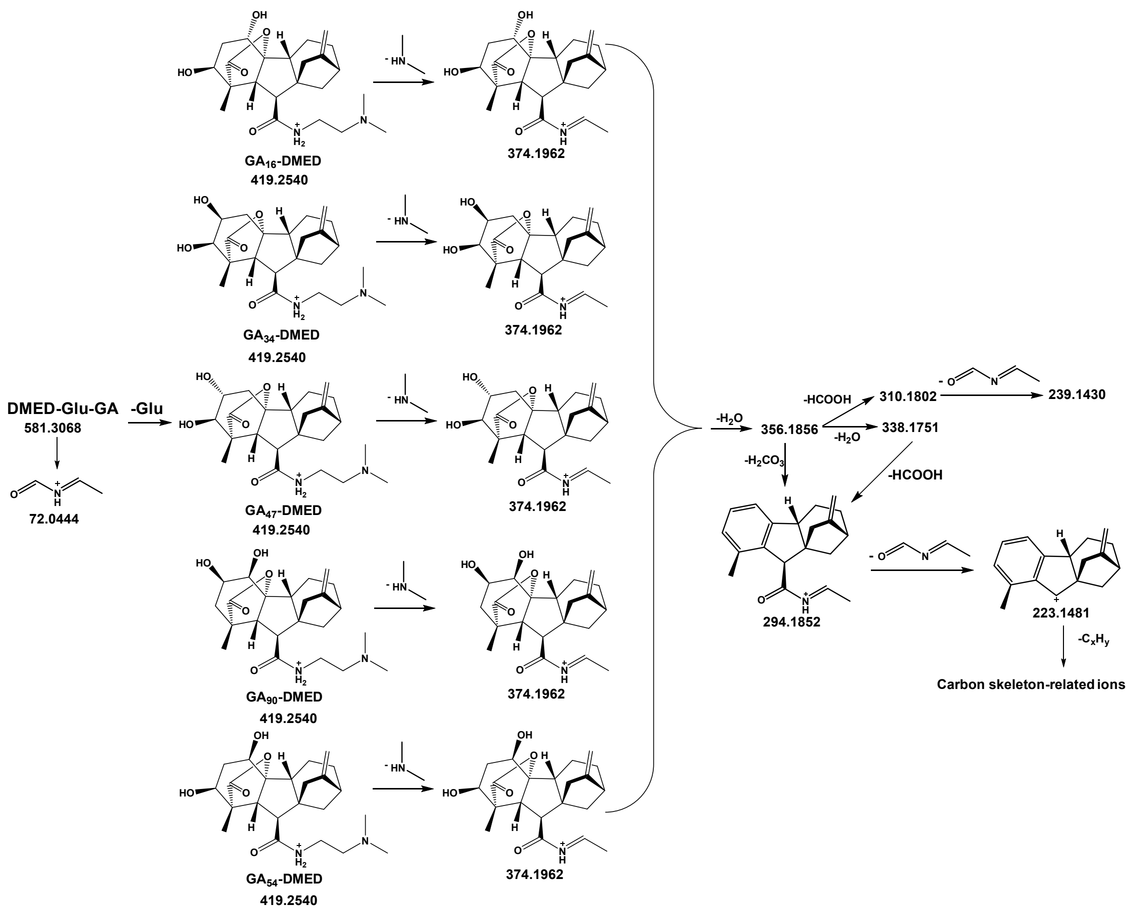

Figure S19. The proposed fragmentation pathways of Glc-GA_{90}-DMED, Glc-GA_{16}-DMED, Glc-GA_{54}-DMED, Glc-GA_{47}-DMED and Glc-GA_{34}-DMED.

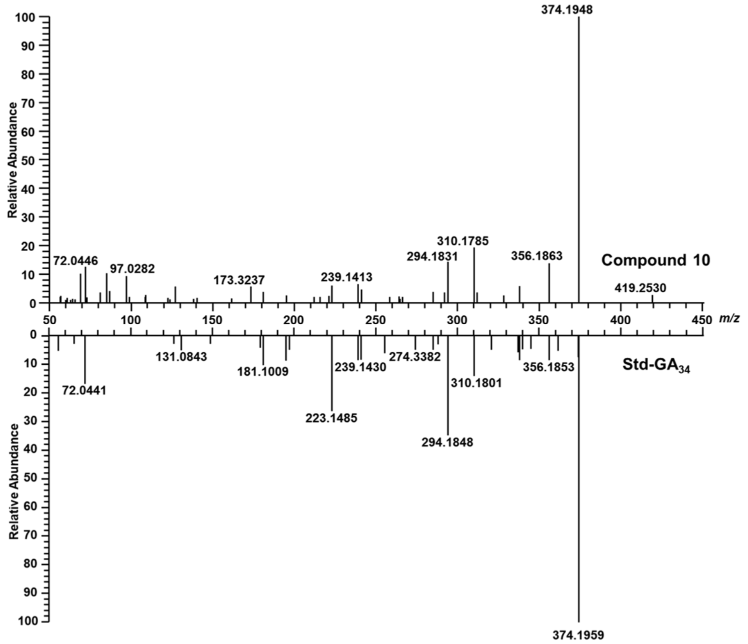

Figure S20. Comparison of HRMS/MS spectra of compound 10 and GA_{34}.

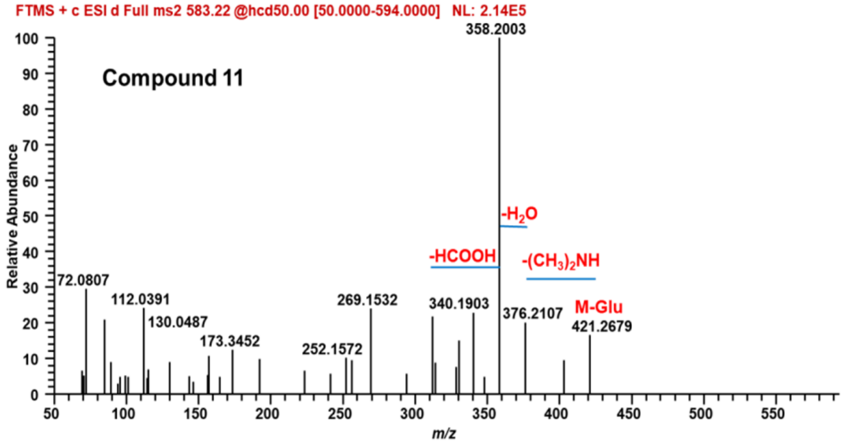

Figure S21. HR MS/MS spectra of compound 11 under positive mode.

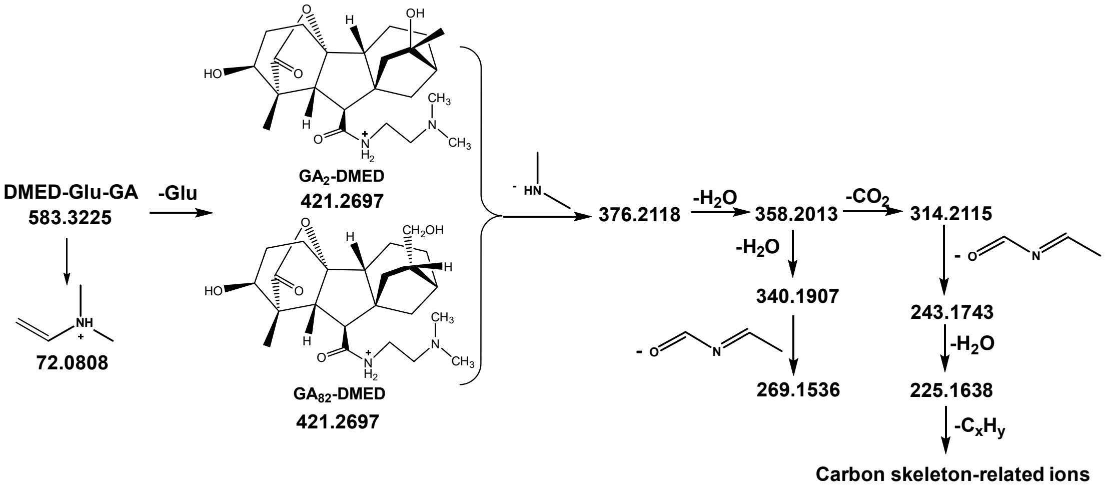

Figure S22. The proposed fragmentation pathways of Glc-GA2-DMED and Glc-GA82-DMED.

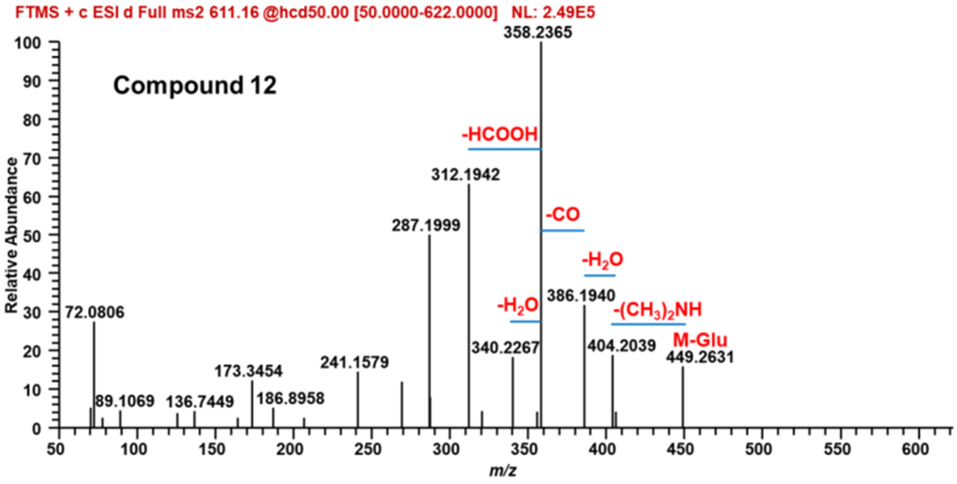

Figure S23. HR MS/MS spectra of compound 12 under positive mode.

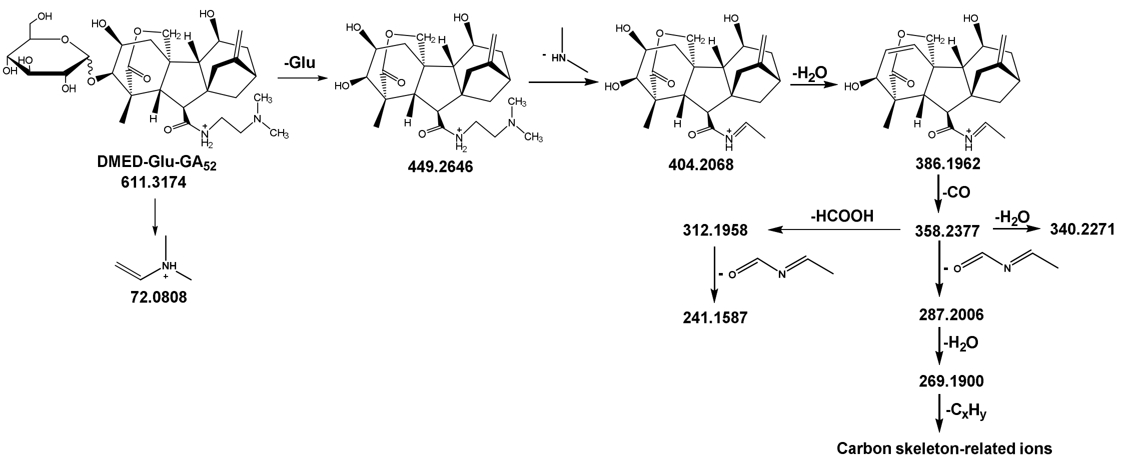

Figure S24. The proposed fragmentation pathways of Glc-GA52-DMED.

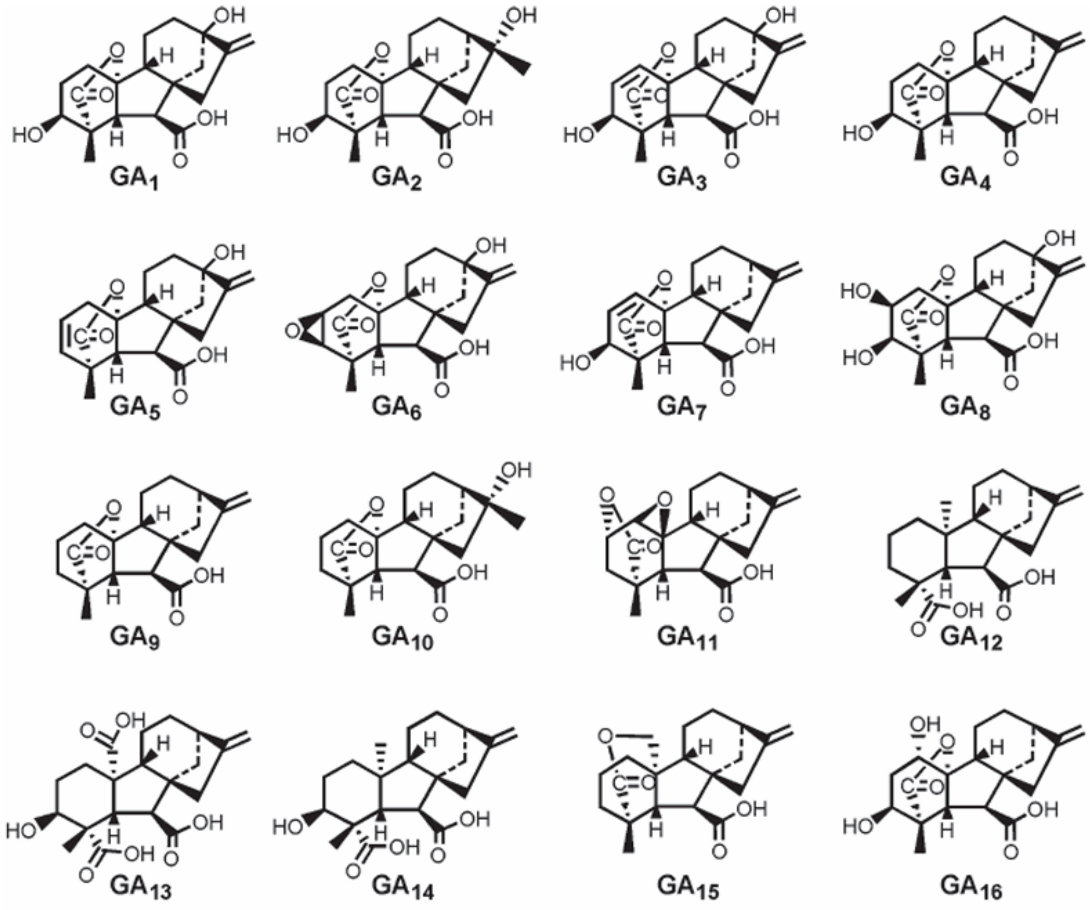

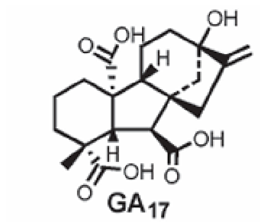
GA17
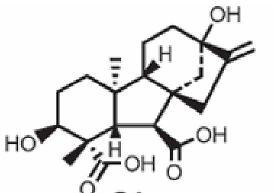
GA18
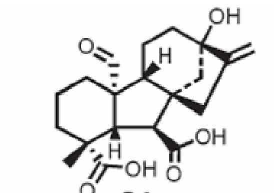
GA19
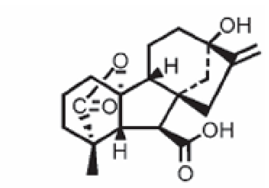
GA20
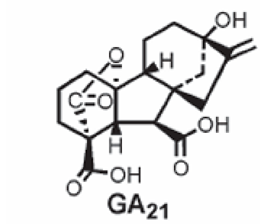
GA21
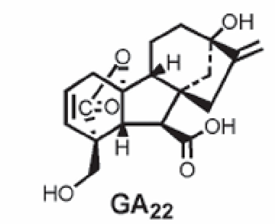
GA22
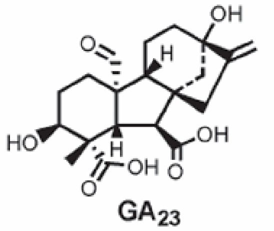
GA23
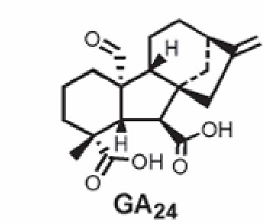
GA24
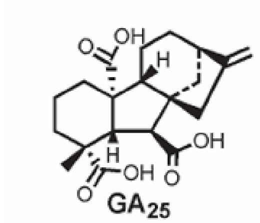
GA25
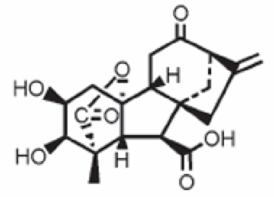
GA26
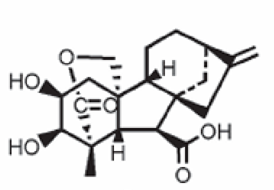
GA27
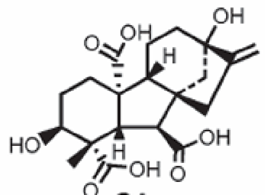
GA28
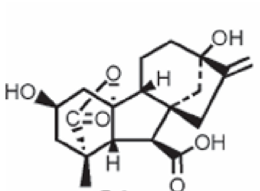
GA29
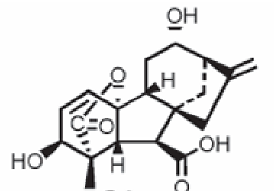
GA30
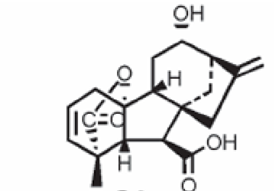
GA31
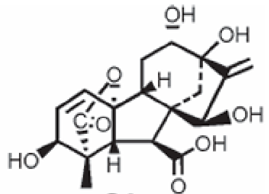
GA32
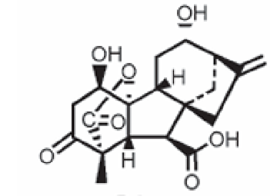
GA33
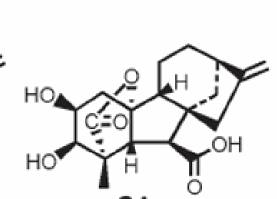
GA34
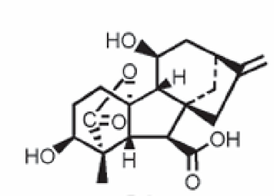
GA35
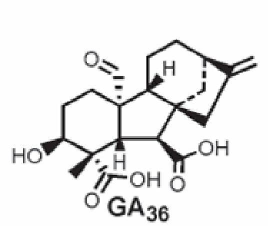
GA36
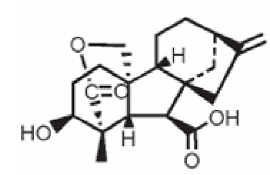
GA37
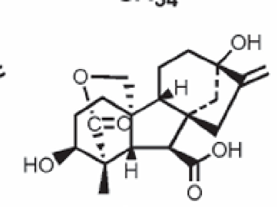
GA38
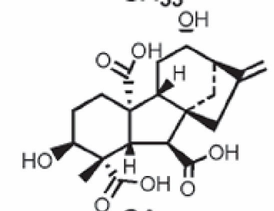
GA39
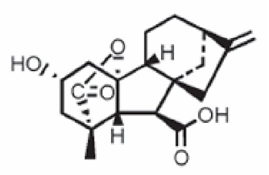
GA40
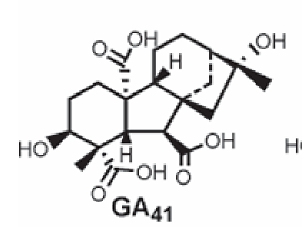
GA41
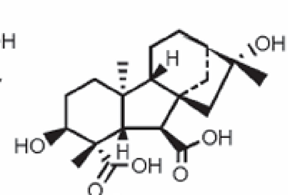
GA42
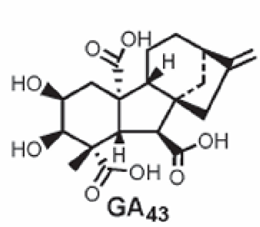
GA43
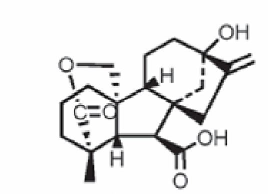
GA44

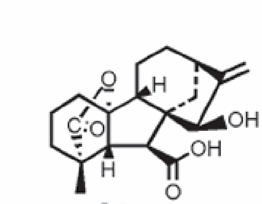
GA$_{45}$

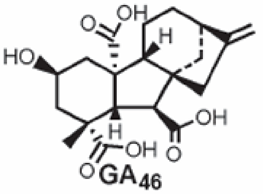
GA$_{46}$

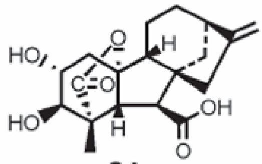
GA$_{47}$

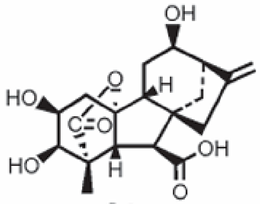
GA$_{48}$

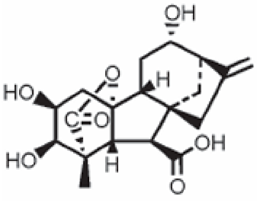
GA$_{49}$

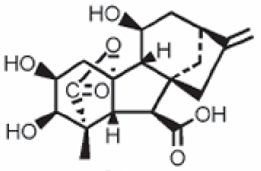
GA$_{50}$

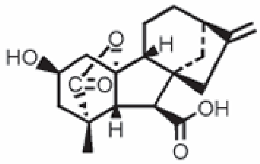
GA$_{51}$

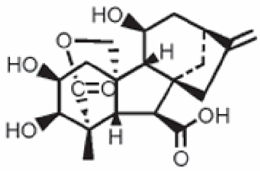
GA$_{52}$

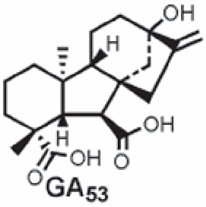
GA$_{53}$

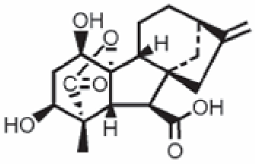
GA$_{54}$

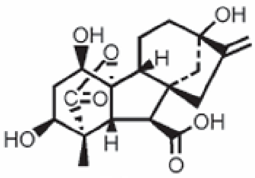
GA$_{55}$

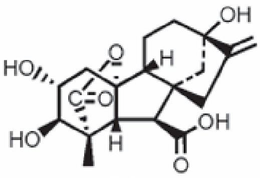
GA$_{56}$

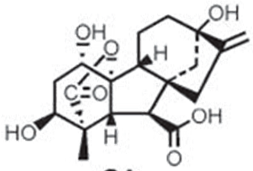
GA$_{57}$

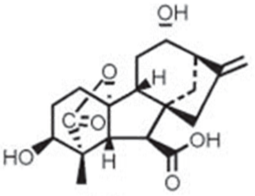
GA$_{58}$

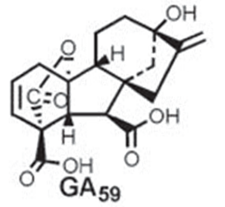
GA$_{59}$

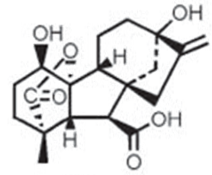
GA$_{60}$

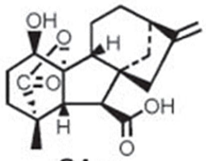
GA$_{61}$

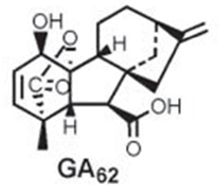
GA$_{62}$

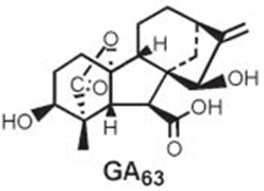
GA$_{63}$

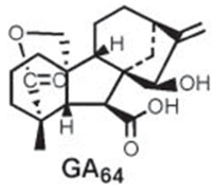
GA$_{64}$

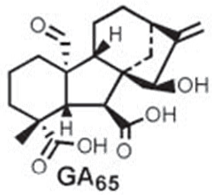
GA$_{65}$

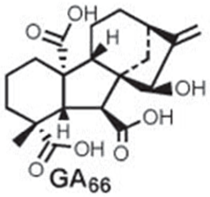
GA$_{66}$

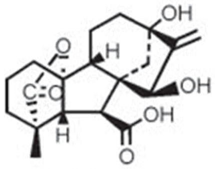
GA$_{67}$

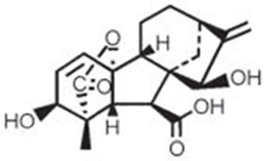
GA$_{68}$

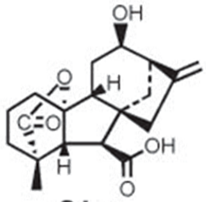
GA$_{69}$

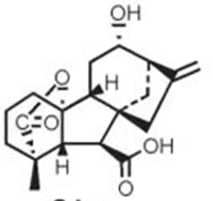
GA$_{70}$

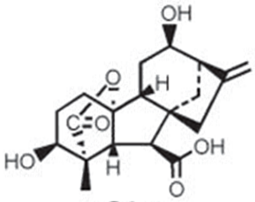
GA$_{71}$

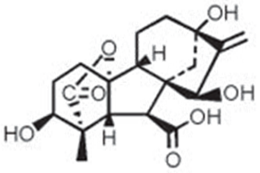
GA$_{72}$

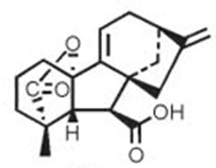
GA_{73}
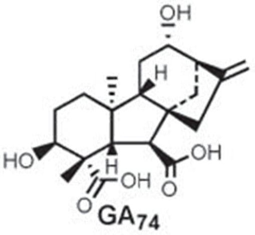
GA_{74}
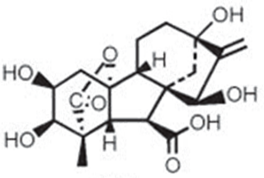
GA_{75}
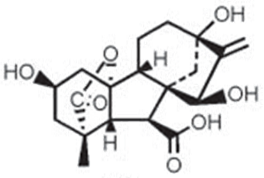
GA_{76}
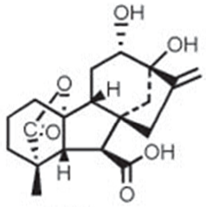
GA_{77}
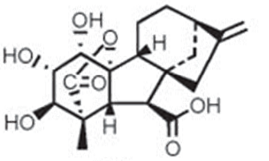
GA_{78}
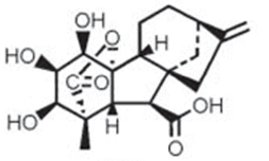
GA_{79}
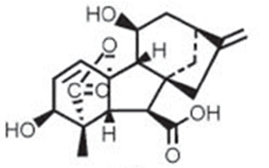
GA_{80}
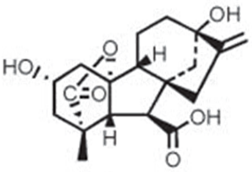
GA_{81}
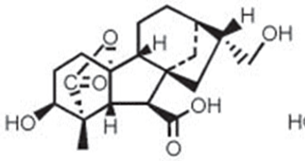
GA_{82}
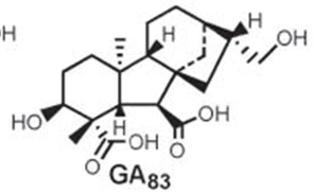
GA_{83}
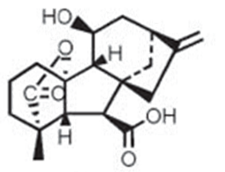
GA_{84}
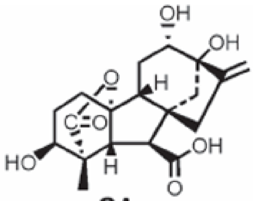
GA_{85}
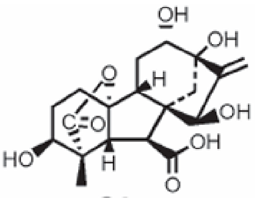
GA_{86}
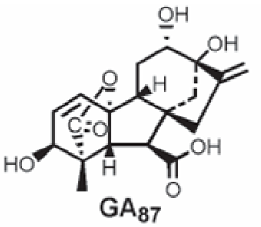
GA_{87}
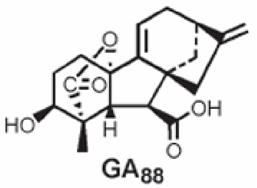
GA_{88}
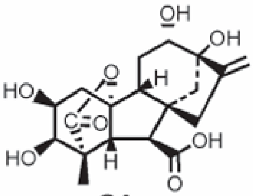
GA_{89}
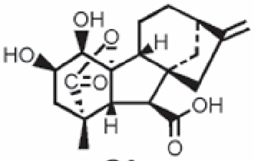
GA_{90}
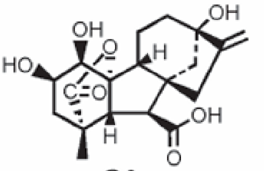
GA_{91}
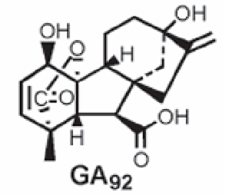
GA_{92}
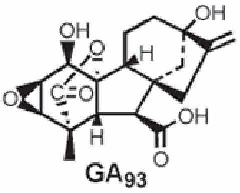
GA_{93}
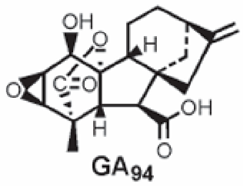
GA_{94}
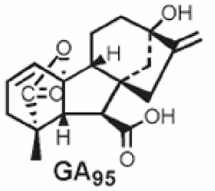
GA_{95}
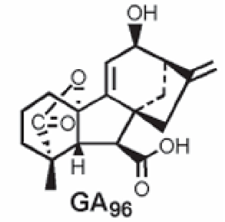
GA_{96}
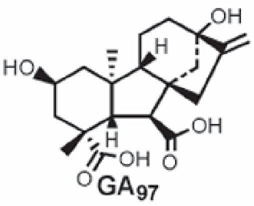
GA_{97}
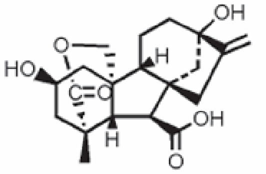
GA_{98}
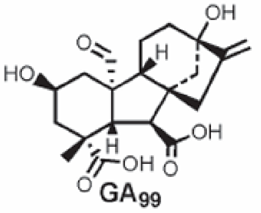
GA_{99}
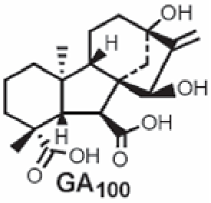
GA_{100}

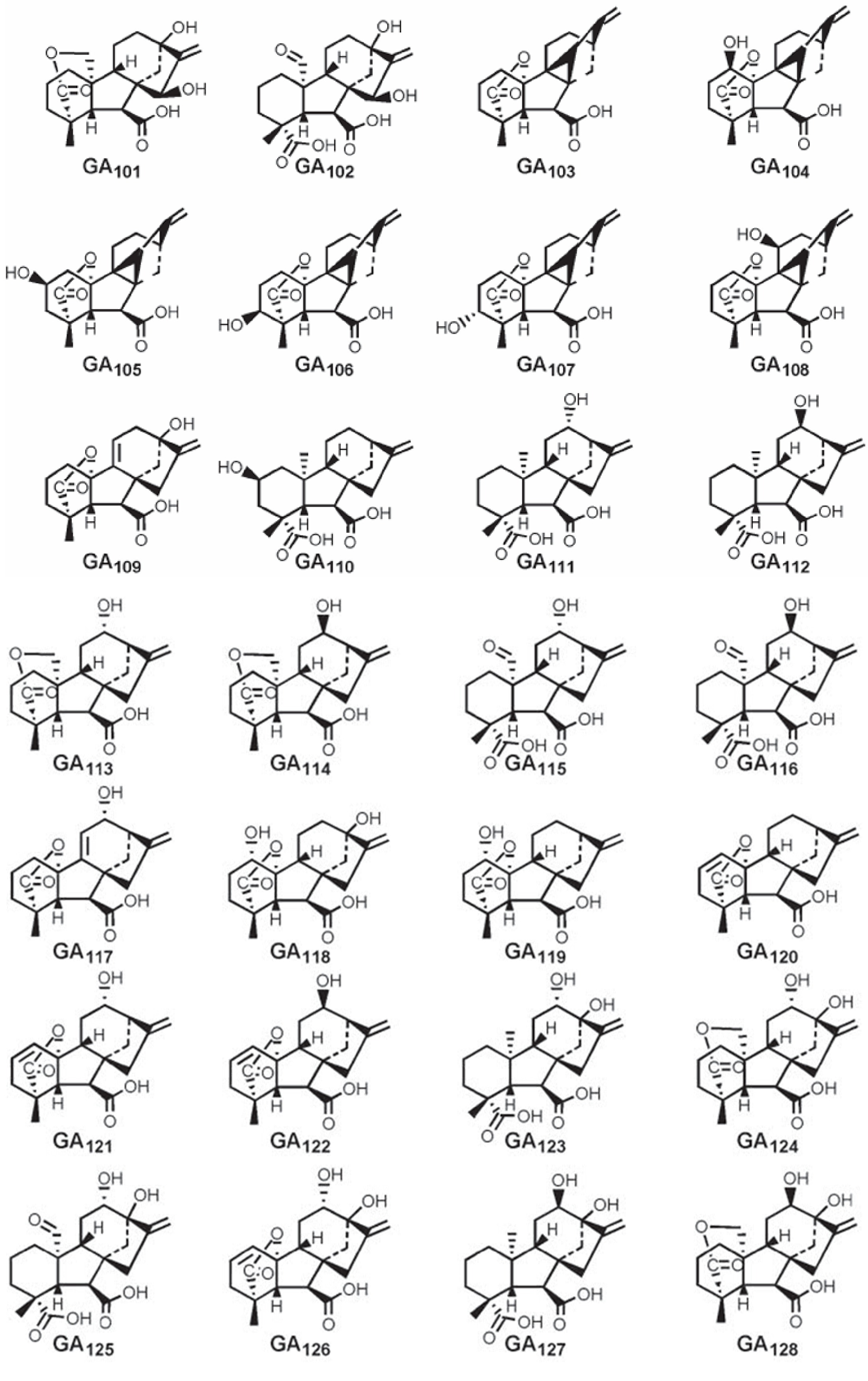

GA101 GA102 GA103 GA104
GA105 GA106 GA107 GA108
GA109 GA110 GA111 GA112
GA113 GA114 GA115 GA116
GA117 GA118 GA119 GA120
GA121 GA122 GA123 GA124
GA125 GA126 GA127 GA128

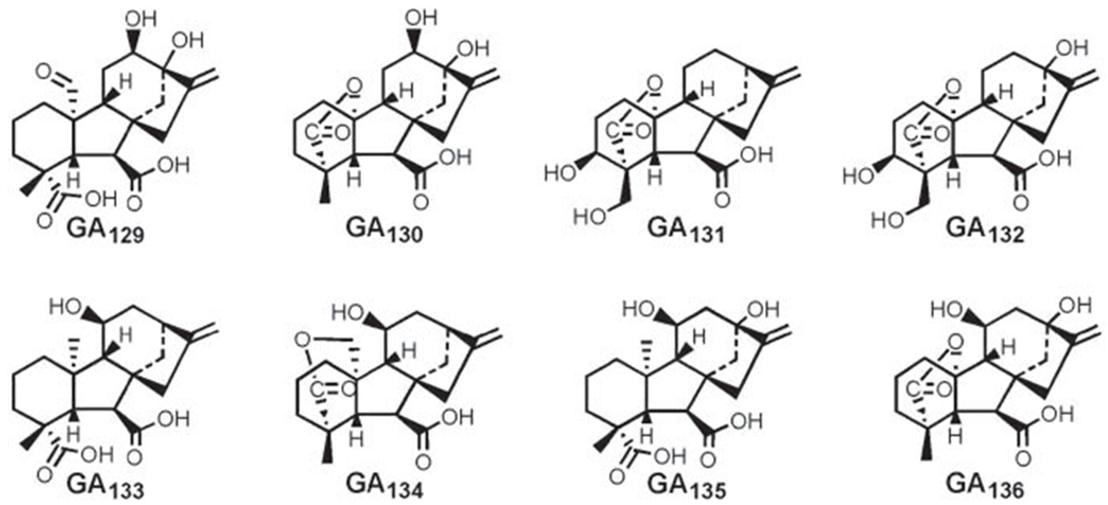

Figure S25. The structures of reported GAs.
